# Characterising public opinion for food-based fiscal policies in the UK

**DOI:** 10.1016/j.socscimed.2026.119269

**Published:** 2026-04-15

**Authors:** John Buckell, Thomas Hancock, Hannah Forde, Lauren Bandy, Henning Jensen, Marcus Keogh-Brown, Ben Amies-Cull, Margarida Bica, Sian Taylor, Owen Thomas, Jane Ward, Mike Rayner, Jessica Renzella, Richard Smith, Laura Cornelsen, Peter Scarborough, Asha Kaur

**Affiliations:** a Health Economics Group, Nuffield Department of Primary Care Health Sciences, University of Oxford, United Kingdom; b Choice Modelling Centre, University of Leeds and Health Economics Group, Nuffield Department of Primary Care Health Sciences, University of Oxford, United Kingdom; c Nuffield Department of Primary Health Care Sciences, University of Oxford, United Kingdom; d Department of Global Health and Development, London School of Hygiene and Tropical Medicine, United Kingdom; e Nuffield Department of Population Health, University of Oxford, United Kingdom; f Patient and Public Involvement Contributor (COPPER: CO-designing for Healthy People and Planet: Food Economic Policy Research Project, NIHR ID: 133887, University of Oxford, UK; g Health Behaviours, Nuffield Department of Primary Health Care Sciences, University of Oxford, United Kingdom; h Faculty of Health and Life Sciences, University of Exeter, United Kingdom; i Department of Public Health, Environments and Society, London School of Hygiene and Tropical Medicine, United Kingdom; j Nuffield Department of Primary Health Care Sciences and Oxford Health Biomedical Research Centre, University of Oxford, United Kingdom

## Abstract

Various fiscal policies have been proposed to incentivise healthy and sustainable diets, with differing attributes and levels. Public support can influence the ability for such policies to be implemented but is currently poorly understood, so two discrete choice experiments (DCEs) were used to measure such preferences among 2020 adults in the UK. The attributes and levels for 192 policy options were identified using a scoping review that comprehensively documented existing or proposed food-based fiscal policies. For health-related taxes and subsidies, options that prioritised protecting the NHS budget were most preferred for all respondents, which held across variation in 9 respondent characteristics. For sustainability taxes, avoiding donating to other countries was a clear priority for all respondents, and again held across 9 respondent characteristics. These results could help to shape UK policy while these methods could be used to understand policy preferences elsewhere.

## 1 Introduction

The imperative for food system reform to promote healthier diets and the environment has been well documented, globally and in the UK (Afshin et al., 2017; Crippa et al., 2021; Food Foundation, 2023). In the UK, healthier and more sustainable diets are relatively expensive (Rao et al., 2013; Hoenink et al., 2024). This is a key determinant of health disparities and a barrier to change (NDNS, 2019; Food Foundation, 2023).

Fiscal policies are seen to be effective measures for altering purchasing habits and changing diets (Afshin et al., 2017; Thow et al., 2014; Pineda et al., 2024). Such policies feature prominently in recent public health policy papers, including taxing sugar-sweetened beverages, taxes on high sugar and salt products, subsidies for fruit and vegetables, financial support for families with children (e.g. food stamps), and hypothecation of food-based taxation (NFS, 2021; WHO, 2022).

Public support eases policy implementation and boosts their effectiveness once implemented (Wright et al., 2017; Pell et al., 2019; Perino and Schwickert, 2023). However, evidence for public support for diet-based fiscal policies is mixed. Mazzocchi et al. (2015) found strong support for healthy eating policies, but that did not translate into tax acceptance. Dieteren et al. (2022) found small increases in sugary drinks taxes were preferred to none, but that no tax was preferred to higher taxes. They found larger support for subsidies on fruit and vegetables. Adams et al. (2021) found strong support for the soft drinks industry levy (SDIL) in the UK. Pechey et al. (2022) and Taillie et al. (2023) studied policies aiming to reduce meat consumption, which pertain to both sustainability and health. The former found that taxation was less popular than many other approaches (e.g. food labelling), though they did not study subsidies. Relatedly, Faccioli et al. (2022) found that combining carbon and health taxes boosts policies’ popularity.

Public support varies for a number of reasons. Mazzocchi et al. (2015) and Dieteren et al. (2022) found considerable heterogeneity in preferences for healthy eating policies across demographics, attitudes, and ideologies; Lancsar et al. (2022) found similar variation in the context of obesity prevention and reduction policies. Pell et al. (2019) note the increased acceptability of food taxes that are hypothecated to health promotion; Cornelsen et al. (2020) found increased support when taxes for sugary drinks are framed as health-related and hypothecated to health promotion. Recent government polling also showed public support for intervention on health, for hypothecation of tax revenues, and for governments supporting individuals on low incomes and families with children (YouGov, 2023). A recent systematic review found that for climate change policies, fairness and effectiveness of the policies were the strongest determinants of public opinion for climate change policies, including taxes (Bergquist et al., 2022). This study also indicates that people’s knowledge, perceptions, and attitudes toward climate change are more impactful on public opinion than their demographic characteristics. Pechey et al. (2022) did not find differences between framing policies towards sustainability or health; nor any associations of policy support and demographic characteristics.

Whilst useful, the available evidence is diffuse across many studies in different countries and settings. It is difficult to triangulate this evidence to inform the development of specific policies. Further, this evidence is not suitable for understanding how different sections of society would prefer policies to a greater or lesser degree, nor for understanding support for a large and varied number of policies, even though these are frequent concerns of policymakers.

To overcome these issues, we provide evidence in three main domains. First, we present overall public preferences for a wide range of policy-relevant attributes of health- and sustainability-related taxes and subsidies derived from a scoping review. This enables us to assess the relative value of individual policy attributes in both heath and sustainability settings. Second, we demonstrate variation of public preferences for those attributes by demographics, attitudes, and diets allowing for a rich analysis of policy acceptability across different sections of society. Third, we combine attribute preferences to examine the overall popularity ranking of 192 policy options that the experiment comprised. Together, this provides a unified and detailed set of findings which can assist policymakers by allowing them to compare directly health and sustainability policies.

Two DCEs focussed on (i) health-based food taxes and subsidies, and (ii) sustainability-based food taxes. A large, national sample of the UK public participated in both. In (i), attributes were the policies’ effects on prices, the foods targeted, the part of society that is the target of the policy, and the generation/use of the policies’ funds. In this DCE we compare a tax that the government has implemented (the UK Soft Drinks Industry Levy, or SDIL), with policies that the government could introduce. That the SDIL is known to the public helps to ground the experiment in reality. An experiment-within-experiment design tested the impact of reminding individuals that subsidies were ultimately paid for by their taxes on preferences for taxes and subsidies. In (ii) the attributes were the taxes’ effects on prices, the target of the taxes, and the spending of the taxes’ proceeds.

Our modelling approach allows us to understand how public opinion varies across demographic characteristics, attitudes, and dietary preferences. This generates nuanced insights that can enable policymakers to align their policies with their target demographics’ preferences.

## 2 Research design and methods

### 2.1 Sampling

Members of the general public that met the eligibility criteria on the basis of age (18 years or older) and residency (UK) were recruited by a survey company (SurveyEngine) between 23rd February 2023 and 9th March 2023.

Of the 8420 individuals invited, 5140 began the survey (Table A1). Of these, survey while 83 were rejected for ineligibility, 955 timed out (survey response time of over 3 hours), 15 were screened out for speeding through (defined as under one third of the average pilot survey time), and 2067 were quotas overfills and so did not proceed beyond the introductory demographic questions. 2020 were used in our final sample.

Quotas for age, gender, ethnicity, and region, based on the UK census, were used to increase the representativeness of the sample, though these were not completely filled. Descriptive statistics for the sample, and a comparison to the UK population, is presented in appendix E. A low income boost was used to ensure that this group was well-represented. In the final sample, 497 (25%) had an income less than £20k per annum (which is in line with the UK ONS’s definition of low income of 60% of the household median).

Some quotas did not reach their target (see Appendix E). To account for this, we used a post-stratification weighting based on age, gender, ethnicity, and region to rebalance the sample. Proportions of these variables were retrieved from the Office for National Statistics. A comparison of weighted and unweighted model results is presented in Appendix C. The results were very similar in the two models.

### 2.2 Discrete choice experiments

Both DCEs were developed based on experimental design principles and health-based good practice guidelines (Reed Johnson et al., 2013; Hensher et al., 2015). Individuals made six choices in a series of choice tasks in both DCEs, in which they were asked “which of these would you choose?” of the two options available. In the health DCE choice tasks, individuals chose between a tax and a subsidy. In the sustainability DCE choice tasks, individuals chose between two taxes because there we did not find a precedent for consumer-focused sustainability subsidies in the literature. Policy effectiveness is being measured indirectly by the impact on prices and not on the resulting behavioural response. The taxes and subsidies in the choice tasks were described by attributes and levels representing different characteristics of taxes and subsidies. A questionnaire collected sociodemographic and attitudinal information alongside the experiment.

### 2.3 Attributes and levels

In the health DCE, four attributes were specified. The number of levels varied for each attribute. Some levels only applied to taxes, and some levels only applied to subsidies due to concerns over the feasibility of scenarios. For example, we assume that the Government would never overtly apply taxes just to individuals on low incomes. Conversely, they may well do so to those on high incomes, achieved through, for example taxing premium products. In the sustainability DCE, three attributes were specified. All attributes for each DCE are summarised in Table 1.

The attributes and levels were based on several sources of evidence: a scoping review of health and environmental taxes and subsidies that identified 753 policies [see full methods in appendix G]; co-production with the Patient and Public Involvement (PPI) members of the project team (n= 2); consultation with subject matter experts in the field (n = 3); and project team members from the Food Foundation (n = 2). The short and long descriptions of the attributes and their levels were modified following PPI feedback on the survey to aid understanding. For example, the target foods level, “sugar-sweetened beverages” was changed to “sugary drinks”. In the health DCE, the level of “sugary drinks tax” was specifically included because such a policy is in place in the UK. Other attribute levels were designed to represent other commonly proposed fiscal policy options. We selected appropriate levels for the taxes and subsidies from the scoping review. Our attribute levels directly mirrored policy recommendations set out in the National Food Strategy (NFS) in the UK (NFS, 2021); for example, the main food groups (with categories of meat, HFSS, fruit and vegetables, and high fibre foods), policies that support people on low incomes, policies that support families with children, and subsidising fresh fruit and vegetables. Recommendation 13 in the NFS is a hypothecated policy, which is an attribute of both DCEs.

A Bayesian D-efficient design was used to generate sets of choice tasks for both experiments (Hensher et al., 2015). Priors were obtained from a pilot study of 116 individuals (not used in the final sample). Individuals were randomised to four blocks of six choice tasks. Each individual answered six choice tasks in each experiment, balancing concerns of learning and respondent fatigue (Hess et al., 2012). Our sample size was substantially in excess of the minimum sample size required for statistical power based on the pilot parameter estimates from a multinomial logit model (de Bekker-Grob et al., 2015). An example of each DCE’s choice task is presented in Fig. 6. In the health DCE, whether the tax option appeared on the left or the right of the choice task was randomised at the level of the individual (i.e., the left-right positions of taxes and subsidies did not change for each individual, but did change across individuals), to enable separation of tax/subsidy preferences from left-to-right bias.

For the health DCE, team members raised concerns that subsidies, which reduce prices, would be preferred to taxes (where prices rise) in all instances. We addressed this in our experiment by imposing an additional layer of randomization in which respondents were assigned to one of two arms prior to the DCE. In one arm, the respondents were exposed to a prime explaining that subsidies are government funded and paid for through taxation. In the other arm, no information was given. This allowed testing of whether preferences for subsidies are mediated by a reminder of government – and ultimately, individual – financial responsibility for them.

### 2.4 Data quality

Prior to the experiment, respondents were given narrative information describing the taxes/subsidies, attributes, and levels. A practice choice scenario aided individuals’ understanding of how the choice scenarios worked, including step-by-step instructions for completing the choice tasks. All information was framed in a way that maximised understanding, drawing on PPI input. In the pilot study (n = 116) we asked respondents to report misunderstandings and/or difficulties, and we adjusted questions as needed in response.

Concerns for online surveys include that respondents may rush through the survey, paying limited attention and not answering accurately. To mitigate these issues, “forced responses” prevented respondents from skipping past questions in the survey; and a minimum time threshold of 2 min, based on pilot data, removed 15 respondents who rushed through. Table A1 shows the total survey responses and which responses were removed.

### 2.5 Statistical analysis

Our modelling approach seeks to explain the choices that participants made in the experiments, which in turn allows us to measure people’s policy preferences. We examine how people’s preferences for the attributes vary according to their demographic characteristics, their attitudes towards climate change, their attitudes towards government intervention, and their diets. Attitudes and diets are understood by drawing on a series of survey questions on each topic; for example, questions such as “do you eat red meat?” were collectively used to measure people’s diets. These were in turn explained by people’s demographic characteristics; for example, whether men or women in our sample ate more meat in their diets. Fig. 7 in the Appendix shows this diagrammatically; Appendix A gives the full survey and all questions. Using these models that explain choices, we can predict probabilities of choosing one policy over another, and thus look at the relative popularity of all policies.

### 2.6 Utility function

In total, 12,120 experimental choices in each experiment were analysed using Multinomial Logistic (Logit) regression models (i.e. 2020 individuals made six choices in each experiment). Based on McFadden (1974), respondents were assumed to maximise their utility in making choices. In this formulation, the individual reconciles their tax/subsidy/attribute preferences for each of the available options and chooses that which maximises their utility; that is, choosing their preferred option in the choice tasks. Respondents’ utility is assumed to be a linearly-additive function,

(1) $${\text{U}}_{\text{nti}}={\text{V}}_{\text{nti}}+\varepsilon_{\text{nti}}=\underset{\text{m}}{\sum }\beta_{\text{m}}\cdot {\text{x}}_{\text{ntim}}+\varepsilon_{\text{nti}}$$

where: U_nti_ is the utility for decision-maker *n* for option *i* in task *t*, comprising deterministic and random utility; V_nti_ is the deterministic component of utility; ε_nti_ is the random component of utility; x_ntim_ is the *m*^th^ attribute-level of option *i*; and β_m_ is the *m*^th^ preference parameter to be estimated. The deterministic component of utility comprises alternative-specific constants (ASC), and attribute levels (continuously coded for level of tax/subsidy, dummy-coded for other attributes), experimental features (behavioural prime for subsidy), and survey artefacts (left-to-right bias).

For the health DCE, we define the deterministic component of utility, V, by experimental attributes and levels:

(2) $$\begin{array}{c} {\text{V}}_{\text{health,nti}}\\ \;={\text{ASC}}_{\text{left-to-right}\;\text{bias}}\cdot \text{left}-\text{hand}\;\text{alternatives}\;\text{in}\;\text{choice}\;{\text{task}}_{\text{health},\text{n}}\\ +{\text{ASC}}_{\text{subsidy}}\cdot \text{subsidy}+{\text{ASC}}_{{\text{subsidy}}_{\text{prime}}}\cdot {\text{subsidy prime}}_{\text{health,n}}\\ +{\text{β}}_{\text{tax}}\cdot {\text{tax}\;\text{income}\;\text{multiplier}\cdot \text{level}\;\text{of}\;\text{tax}}_{\text{health,nti}}\\ +{\text{β}}_{\text{subsidy}}\cdot {\text{subsidy}\;\text{income}\;\text{multiplier}\cdot \text{level}\;\text{of}\;\text{subsidy}}_{\text{health,nti}}\\ \;+\sum_{\text{L}-1=1}{\text{β}}_{\text{target}\;\text{of}\;\text{subsidy}}\cdot {\text{target}\;\text{of}\;\text{the}\;\text{subsidy}}_{\text{health,nti}}\\ +\sum_{\text{L}-1=3}{\text{β}}_{\text{target}\;\text{of}\;\text{tax}}\cdot {\text{target}\;\text{of}\;\text{the}\;\text{tax}}_{\text{health,nti}}\\ \;+\sum_{\text{L}-1=4}{\text{β}}_{\text{who}\;\text{the}\;\text{policy}\;\text{affects}}\cdot {\text{who}\;\text{the}\;\text{policy}\;\text{affects}}_{\text{health,nti}}\\ \;+\sum_{\text{L}-1=4}{\text{β}}_{\text{where}\;\text{the}\;\text{money}\;\text{goes}\;\text{comes}\;\text{from}}\cdot {\text{where}\;\text{the}\;\text{money}\;\text{goes}\;\text{comes}\;\text{from}}_{\text{health,nti}} \end{array}$$

For the sustainability DCE, we define the deterministic component of utility, V, by experimental attributes and levels:

(3) $$\begin{array}{c} {\text{V}}_{\text{sus,nti}}\\ \begin{array}{c} ={\text{ASC}}_{\text{left-to-right}\;\text{bias}}\cdot \text{left}-{\text{hand}\;\text{alternative}\;\text{in}\;\text{choice}\;\text{task}}_{\text{sus}}\\ +\beta_{\text{tax}}\cdot \text{tax}\;\text{income}\;\text{multiplier}\cdot {\text{level}\;\text{of}\;\text{tax}}_{\text{sus,nti}}\\ +\sum_{\text{L}-1=3}\beta_{\text{target}\;\text{of}\;\text{tax}}\cdot {\text{target}\;\text{of}\;\text{the}\;\text{tax}}_{\text{sus,nti}}\\ +\sum_{\text{L}-1=3}\beta_{\text{where}\;\text{the}\;\text{money}\;\text{goes}}\cdot {\text{where}\;\text{the}\;\text{money}\;\text{goes}}_{\text{sus,nti}} \end{array} \end{array}$$

Where left-hand alternative in choice task_n_ is an indicator for if the alternative was on the left-hand side in choice tasks (cf. Fig. 6). It varies by individual for the health DCE because the left-right position of taxes and subsidies was changed across individuals (but constant within individuals); this was not the case for the sustainability DCE where both alternatives were unlabelled taxes, “tax 1” and “tax 2”. $\text{tax}\;\text{income}\;\text{multiplier}={\left(\frac{{\text{income}}_{\text{n}}}{\tilde{\text{income}}}\right)}^{\lambda_{\text{tax}}}$ and $\text{subsidy}\;\text{income}\;\text{multiplier}={\left(\frac{{\text{income}}_{\text{n}}}{\tilde{\text{income}}}\right)}^{\lambda_{\text{subsidy}}}$; income_n_ is that individual’s $\tilde{\text{income}}$ is the median income in the sample; λ_tax_ and λ_subsidy_ are income elasticities for taxes and subsidies, respectively, to be estimated. *L* is the total number of levels for that attribute. Since the attributes are dummy coded, there are *L-1* parameters estimated. To illustrate, for ∑_L−1=3_β_where the money goes_ are in the sustainability DCE, “general taxation” is the reference category, and we estimate three parameters of β_chepaer low impact foods_, β_green projects_, and β_donate to other countries_. In Table 1, the reference level for each attribute in both DCEs is shown.

To enable comparisons across experiments that are scale-free, and to ease interpretation of the parameter estimates, we estimate models in the willingness-to-be-taxed (WTbT) space (Train and Weeks, 2005). Thus, parameter estimates directly measure marginal rates of substitution between the attribute at hand and the level of tax, and can be interpreted as the value of each parameter expressed in terms of the amount that individuals are willing to be taxed. Taking the sustainability DCE as an example, we have,

(3a) $$\begin{array}{c} {\text{V}}_{\text{sus,nti}}+\varepsilon_{\text{sus,nti}}={\text{ASC}}_{\text{left-to-right}\;\text{bias}}\cdot \text{left}-\text{hand}\;\text{alternative}\;\text{in}\;\text{choice}\;\\ \text{task}+\beta_{\text{tax}\;}\cdot \text{tax}\;\text{income}\;\text{multiplier}\cdot \\ ({\text{level}\;\text{of}\;\text{tax}}_{\text{sus,nti}}+\sum_{\text{L}-1=3}\beta_{\text{target}\;\text{of}\;\text{tax}}\\ \cdot {\text{target}\;\text{of}\;\text{the}\;\text{tax}}_{\text{sus,nti}}+\sum_{\text{L}-1=3}\beta_{\text{where}\;\text{the}\;\text{money}\;\text{goes}\;}\\ \cdot {\text{where}\;\text{the}\;\text{money}\;\text{goes}}_{\text{sus,nti}})+\varepsilon_{\text{sus,nti}} \end{array}$$

Assuming a type-I extreme value error distribution on the error term results in a multinomial logit (MNL) model for estimating choice probabilities:

(4) $${\text{P}}_{\text{health},\text{nti}}=\frac{\text{exp}\left({\text{V}}_{\text{health},\text{nti}}\right)}{\sum_{\text{j}=1}^{\text{J}}\text{exp}\left({\text{V}}_{\text{health},\text{ntj}}\right)},$$

(5) $${\text{P}}_{\text{sus},\text{nti}}=\frac{\exp\left({\text{V}}_{\text{sus,nti}}\right)}{\sum_{\text{j}=1}^{\text{J}}\exp\left({\text{V}}_{\text{sus,ntj}}\right)}.$$

Where *J* is the total number of alternatives.

### 2.7 Preference heterogeneity

Heterogeneous preferences were modelled deterministically and randomly. We first model deterministic heterogeneity using information on participants in the survey. We then test if we can capture any remaining heterogeneity using random parameters. It is preferable where possible to model heterogeneity deterministically, because we can link variation in preferences to individual characteristics, which is useful information for interpreting findings. We note that post model selection procedures, such as selection of interactions, are a threat to valid inferences being drawn. However, in the case of understanding preferences, we need to balance this with confounding. For example, if preferences are linked to higher education, and higher education is linked to female gender, if we only interact gender with preferences, we would (assuming no effect of gender) pick up the correlation with higher education and potentially misattribute that preference to gender. Hence, the convention in the choice modelling literature is to test down to avoid such confounding. For deterministic heterogeneity, ASCs and the attribute levels were interacted with individuals’ characteristics (age, gender, ethnicity, education, urbanicity).

In addition to individuals’ characteristics, a series of questions were collected on: climate change, government intervention on health and climate change, and diets. The full list of survey questions is presented in Appendix A; Appendix F gives the rationale for the use of latent variables.

Based on a factor analysis and PPI advice, three latent variables were defined. The factor analysis helps to identify correlations in the variables. Combinations of variables were sense-checked with study PPI members. The first measured a climate change attitude, where a higher value meant the individual believed more strongly in climate change. This comprised five indicators (i.e., responses to survey questions): belief in climate change, worry about climate change, responsibility for climate change, whether participants would pay for environmentally friendly products, and whether the government should intervene on climate change. The second measured meat-free diets, where a higher score meant the individual ate less meat and fewer dairy products. This comprised six indicators: vegan/vegetarian status, whether the individual ate red meat, white meat, fish, dairy, or none of those. The third measured attitudes towards government intervention, where a higher score meant the individual believed more strongly that the government should intervene in markets. This comprised five indicators: whether the government should intervene in health, whether the government should intervene for the environment, whether participants would pay for environmentally friendly products, whether the government should tax sugary drinks, whether the government should tax sugary drinks and spend the proceeds on improving health.

Random preference heterogeneity was modelled using mixing distributions (Train, 2009). The parameters are treated as normal distributions each with a mean and standard deviation to be estimated. The exception is for the tax sensitivities, for which we used a loguniform mixing distribution for the following reasons. First, we wanted a specification that imposed unidirectional preferences to align our model with economic theory (individuals should prefer lower taxes to higher taxes). Second, we wanted a specification that imposed unidirectional preferences for tax to enable interpretation of the WTbT coefficients elsewhere in the model (i.e. the denominator of the WTbT calculation does not cross zero). Third, the fit of the loguniform was preferred to the lognormal distribution that was tested. The distributions were assumed to be uncorrelated.

Combining deterministic and random heterogeneity, taking β_UK green projects_ as an example, we have,

(6) $$\begin{array}{c} \beta_{\text{UK}\;\text{green}\;\text{projects}}^{\left(\text{n}\right)}={\text{mean}}_{\text{UK}\;\text{green}\;\text{projects}}+{\text{s}.\text{d}.}_{\text{UK}\;\text{green}\;\text{projects}}\cdot \left(\theta_{\text{UK}\;\text{green}\;\text{projects,n}}\right)\\ +{\text{mean}\;\text{shift}}_{\text{UK}\;\text{green}\;\text{projects}}\cdot {\text{Z}}_{\text{n}}+\lambda_{\text{cc}\;\text{belief}}\cdot {\text{LV}}_{\text{cc,n}}\\ +\lambda_{\text{meat}\;\text{free}}\cdot {\text{LV}}_{\text{mf,n}}+\lambda_{\text{gov}\;\text{int}}\cdot {\text{LV}}_{\text{gov,n}} \end{array}$$

where mean_UK green projects_ is the mean of a normal preference distribution for tax revenue being spent on UK-based environmentally friendly products (as opposed to general taxation). The standard deviation of the normal distribution given by s.d._UK green projects_, where θ_UK green projects,n_ ~ N (0, 1) is a standard normal distribution. Z_n_ is a vector of individual characteristics (age, gender, etc.) and mean shift_UK green projects_ are pa-rameters measuring differential preferences by individual characteristics, to be estimated (i.e. whether there is a shift in the mean of the preference distribution associated with each characteristic). LV_cc,n_, LV_mf,n_, and LV_gov,n_ are latent variables for climate change, meat-free diets, and government intervention, respectively; λ_cc belief_, λ_meat free_, and λ_gov int_ measure how each latent variable correlates with attribute (marginal) utilities.

In the case of the loguniform distribution for tax sensitivities, the specification is similar but with three differences. The mean of the normal distribution is replaced by a bound (i.e., a shift away from zero) on a uniform distribution, bound_tax_. The standard deviation of the normal distribution is replaced with a range parameter multiplied by draws from a uniform distribution, range_tax_⋅(θ_tax_), where θ_tax_ ~ U(0, 1). The loguniform is achieved with a simple transformation of the uniform, $-{\text{e}}^{\left(\beta_{\text{tax}}\right)}$.

We next turn to specification of the hybrid choice aspects of the model (for an overview of this approach, see Abou-Zeid and Ben-Akiva (2024)). Indicators of latent variables took one of three forms: binary variables (e.g., yes/no for “Do you believe in climate change?”), ordered variables (e.g., Likert scale of 1 to 5 for “How worried are you, if at all, about climate change?”), or continuous variables (e.g., scalar response from 0 to 10 for “To what extent do you feel a personal responsibility to try to prevent climate change from worsening?”). Taking these three as examples of the climate change latent variable indicators, they are specified in measurement equations to estimate the relationship between the latent variable and its indicator variables.

#### 2.7.1 Binary variables use a logit

(7) $${\text{P}}_{\text{cc}\;\text{belief}}=\frac{\exp\left(\delta_{\text{cc}\;\text{belief}\;}+\zeta_{\text{cc}\;\text{belief}}\cdot {\text{LV}}_{\text{cc,n}}\right)}{1+\exp\left(\delta_{\text{cc}\;\text{belief}}+\zeta_{\text{cc}\;\text{belief}}\cdot {\text{LV}}_{\text{cc,n}}\right)}$$

Where δ_cc belief_ is a constant term to be estimated, LV_cc,n_ is the latent variable for climate change, and ζ_cc belief_ measures the relationship between the latent variable and the response to the question.

#### 2.7.2 Ordered variables use an ordered logit

(8) $$\begin{array}{c} {\text{P}}_{\text{cc}\;\text{worry}}=\sum_{\text{s}=1}^{\text{S}}\delta \left({\text{I}}_{\text{cc}\;\text{worry}}==\text{s}\right)[\frac{\exp\left(\tau_{\text{cc}\;\text{worry,s}}-\zeta_{\text{cc}\;\text{worry}}\cdot {\text{LV}}_{\text{cc,n}}\right)}{1+\exp\left(\tau_{\text{cc}\;\text{worry,s}}-\zeta_{\text{cc}\;\text{worry}}\cdot {\text{LV}}_{\text{cc,n}}\right)}\\ -\frac{\exp\left(\tau_{\text{cc}\;\text{worry,s-1}}-\zeta_{\text{cc}\;\text{worry}}\cdot {\text{LV}}_{\text{cc,n}}\right)}{1+\exp\left(\tau_{\text{cc}\;\text{worry,s-1}}-\zeta_{\text{cc}\;\text{worry}}\cdot {\text{LV}}_{\text{cc,n}}\right)}] \end{array}$$

Where τ_cc worry,s_ are estimated threshold parameters for threshold s of the climate change worry indicator, I_cc worry_. LV_cc_ is the latent variable for climate change, and ζ_cc worry_ measures the relationship between the latent variable and the response to the question.

#### 2.7.3 Continuous variables use a linear model

(9) $$\begin{array}{c} {\text{P}}_{\text{cc}\;\text{responsibility}}=\frac{1}{\sqrt{2\pi \sigma_{\text{cc}\;\text{responsibility}}^{2}}}\cdot \exp\\ \;-\left(\frac{{\text{I}}_{\text{cc}\;\text{responsibility}}-\bar{{\text{I}}_{\text{cc}\;\text{responsibility}}}-\zeta_{\text{cc}\;\text{responsibility}}\cdot {\text{LV}}_{\text{cc,n}}}{2\sigma_{\text{cc}\;\text{responsibility}}^{2}}\right) \end{array}$$

Where using the demeaned variable (operationalised by subtracting $\bar{{\text{I}}_{\text{cc}\;\text{responsibility}}}$) avoids estimating a constant, and $\sigma_{\text{cc}\;\text{responsibility}}^{2}$ is the estimated error variance. LV_cc_ is the latent variable for climate change, and ζ_cc responsibility_ measures the relationship between the latent variable and the response to the question.

Next, a structural equation for each latent variable measures the relationship between latent variables and individual characteristics. Taking the climate change latent variable as an example,

(10) $${\text{LV}}_{\text{cc,n}}=\gamma_{\text{cc}}\cdot {\text{Z}}_{\text{n}}+\xi_{\text{n,cc}}$$

where Z_n_ are individual characteristics, γ_cc_ capture the relationships between the latent variable for climate change and individual characteristics. ξ_n,cc_ captures random heterogeneity following a standard normal distribution with zero mean and unit variance, ξ_n,cc_ ~ N(0, 1).

#### 2.7.4 Joint estimation

With the addition of the latent variables, an issue arises if both choice experiments are analysed separately, which is that estimates for the latent variables differ, meaning that the implied attitudes of individuals also differ. This would be incorrect because the same individuals have the same attitudes and only gave a single response to each of the attitudinal/lifestyle questions. To avoid this, the models for each DCE, the measurement equations, and the structural equations are combined in a single system of equations. This specifies a single set of the three latent variables, measurement equations, and structural equations that feed simultaneously into both choice models for both DCEs. This ensures that people’s attitudes are the same across both DCEs, which is behaviourally appropriate. In this setup, the three latent variables are used only once to explain each set of their respective indicators. Each latent variable is explained only once by individuals’ characteristics. Each latent variable enters both utility functions to explain how the attitudes are related to tax and subsidy preferences for health and for sustainability. Fig. 7 shows a schematic of the system of equations.

Given both the mixing distributions and error terms on the latent variables, the resulting likelihood function no longer has a closed form solution. To facilitate estimation, we use simulations and integrate over both the mixing distributions and the latent variables to approximate the likelihood function.

(11) $$\text{SLL}={\text{w}}_{\text{n}}\sum_{\text{nt}}\text{log}\int_{\beta }\int_{\text{LV}}{\text{P}}_{\text{health,ni}}{\text{P}}_{\text{sus,ni}}{\text{P}}_{\text{binary,n}}{\text{P}}_{\text{ordered,n}}{\text{P}}_{\text{linear,n}}\phi (\zeta )\text{m}(\beta )\text{d}\beta \text{dLV}$$

where SLL is the simulated log-likelihood. w_n_ is an individual-specific, post-stratification weighting, based on age group, gender, ethnicity, and region. These were calculated by raking using the package survey in R. P_health,ni_ and P_sus,ni_ are the choice probabilities for the health and sustainability DCEs. P_binary,n_, P_ordered,n_, and P_linear,n_ are probabilities for the measurement equations of binary, ordered and continuous indicator variables. 500 draws were taken using the Modified Latin Hypercube Sampling algorithm (Hess et al., 2006). Robust standard errors, taking account of the panel structure in the data, are computed using the sandwich estimator. All models were estimated using simulated maximum likelihood with Bunch et al. (1993), BGW, optimisation in the Apollo package for R (Hess and Palma, 2019).

### 2.8 Testing and sensitivity analyses

In initial analyses, we estimated specifications where the full set of interaction terms of individual characteristics and attributes was specified for each attribute. Assumptions on mixing distributions were tested (normal and uniform) and the preferred were selected. We then conducted several rounds of refinement in which non-significant interaction terms were removed. An initial specification of 319 parameters was pared down to 159 parameters.

A model that used vegetarian and vegan variables separately was tested against a model that combined both into a single indicator; the latter was preferred.

Several specifications of mixing distribution for the tax coefficient were tested. The loguniform was preferred in terms of model fit.

A model without weights was tested against the weighted model. Few, if any, differences in estimates were evident, results are shown in Appendix C.

### 2.9 Simulations

After estimation, we used the fitted model to compute the probabilities of individuals choosing various fiscal policies. Based on the estimated parameters, we simulated choices for all combinations of attribute levels (i.e., the possible taxes and subsidies set out by attribute level combinations in the experimental design). In each simulation, we set the attribute values in the data to a specific combination and computed probabilities using the model. The model is based on two alternatives which allows pairwise comparisons of any combination of fiscal policies that were set out in the experiment. By taking any one of these and using it as a comparator (i.e., benchmark), we can rank the entire set of policies used in the experiment relative to that comparator.

For the health DCE, the obvious comparator is the sugary drinks tax (SDIL) implemented in 2018. If we set the attributes for an alternative to be those of the SDIL, then based on equation (2), we would have V_SDIL_. We can then compare any other policy to that by changing the attribute values and computing a new V. If we define the policy of interest V_health policy_, then we can compute the probability that this is preferred to the SDIL by using equation (4), such that,

(12) $${\text{P}}_{\text{health}\;\text{policy}}=\frac{\exp\left({\text{V}}_{\text{health}\;\text{policy}}\right)}{\sum_{j=1}^{J}\exp\left({\text{V}}_{\text{health,nj}}\right)}=\frac{\exp\left({\text{V}}_{\text{health}\;\text{policy}\;}\right)}{\exp\left({\text{V}}_{\text{health}\;\text{policy}}\right)+\exp\left({\text{V}}_{\text{SDIL}}\right)}$$

It follows that,

(13) $${\text{P}}_{\text{SDIL}}=\frac{\exp\left({\text{V}}_{\text{SDIL}}\right)}{\exp\left({\text{V}}_{\text{SDIL}}\right)+\exp\left({\text{V}}_{\text{SDIL}}\right)}=\frac{1}{2}=0.5$$

That is, the probability of choosing the SDIL compared to the SDIL is exactly 50% since it is the same thing. So, if a policy is more popular than the SDIL, V_health policy_ > V_SDIL_, and the probability is higher than 0.5. If a policy is less popular than the SDIL, V_health policy_ < V_SDIL_, and the probability is lower than 0.5.

The procedure for the sustainability DCE is exactly the same, except the choice of comparator is arbitrary because none of the policies are in place in the real world. Note, however, that this choice is irrelevant because in a choice model, only relative differences matter for identification. The implication of this is that the order of predicted probabilities of the taxes is independent of the choice of comparator.

## 3 Results

### 3.1 Public preferences for attributes of taxes and subsidies

**Fig. 1:**
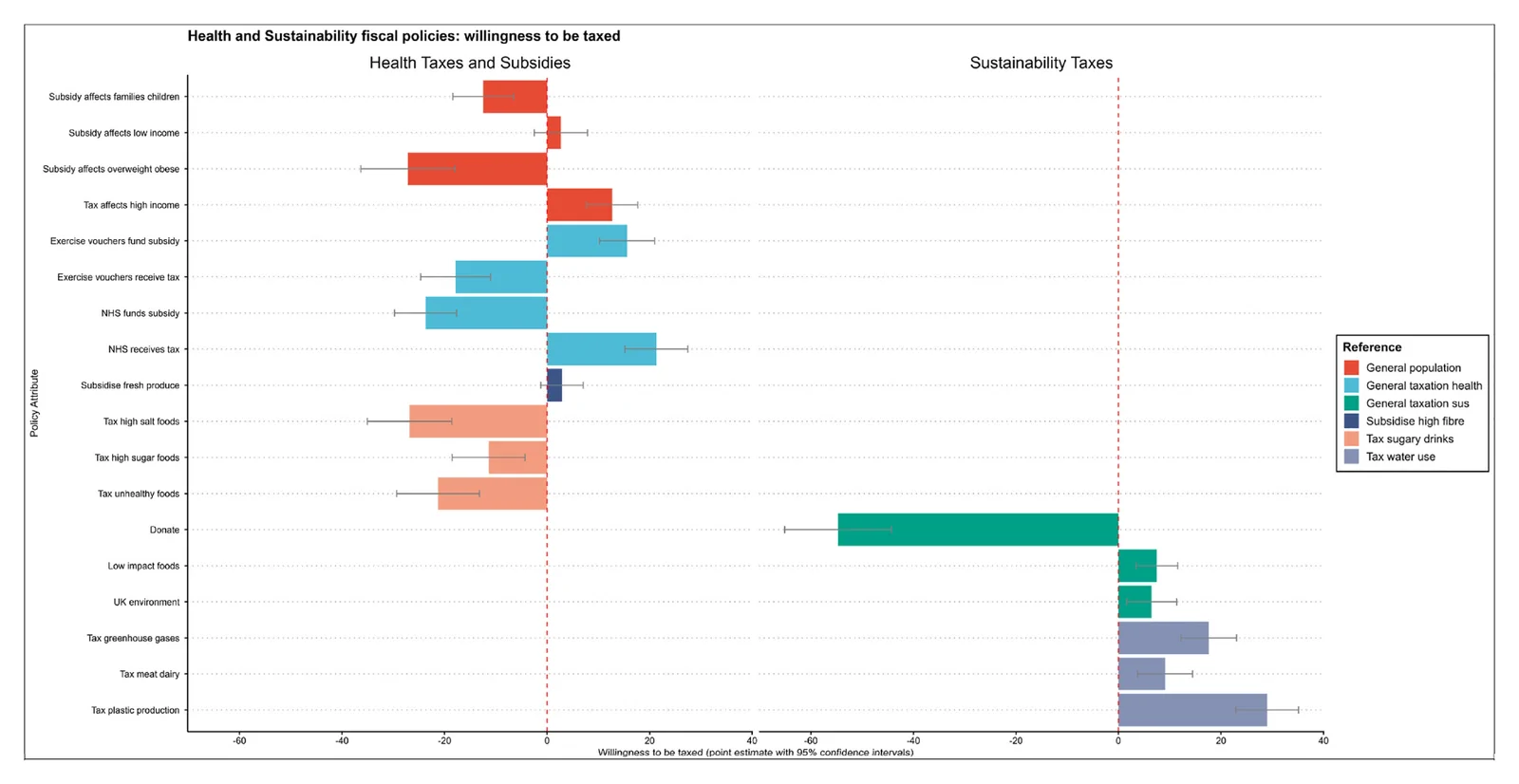
Overall preferences for fiscal policy attributes. Preferences are expressed in willingness to be taxed (WTbT), i.e. respondents’ value of the attribute level, relative to its reference, in terms of the amount of tax. 95% confidence intervals are computed for this ratio using the delta method. Values indicate the amount of extra tax respondents were willing to be taxed (or reductions in prices for negative values) on goods for that attribute, relative to its reference. For example, for health policies, respondents in the sample were willing to be taxed 22% more if the proceeds of the tax were hypothecated to the NHS rather than into general taxation.

Preferences for policies’ attributes are presented in Fig. 1. These are expressed in terms of respondents’ willingness-to-be-taxed (WTbT) for each attribute, for two reasons. First, it allows the expression of preferences in terms of an amount of tax that is tangible (as opposed to the standard latent scale of respondents’ utility, which is intangible). Second, it enables direct comparisons across the experiments (which is inappropriate for utilities because of scale differences across the models that do not apply to scale-free measures such as WTbT). In this setup, parameter estimates directly measure marginal rates of substitution between the attribute at hand and the level of tax, and can be interpreted as the value of each parameter, compared to the base level, expressed in terms of the tax percentage.

Protecting or adding to NHS funding was the most preferred attribute (WTbT: 22.01%, 95%CI: 21.62% to 22.41%). This means that, for a given tax, respondents could be taxed 22% more and be equally satisfied if the proceeds went specifically to the NHS rather than to general taxation. Conversely, taking from NHS budgets for food subsidies was disliked (WTbT: −24.78%, 95%CI: −24.91% to −24.65%). That is, if subsidies were raised from the NHS budget they should be 24% lower than the same tax raised from general taxation. Taxing sugary drinks was preferred amongst the different food options presented (i.e. taxes could be higher on sugary drinks): preferences were negative for taxing high salt foods (WTbT: −29.39%, 95%CI: −29.64% to −29.13%), unhealthy foods (WTbT: −21.70%, 95%CI: −22.01% to −21.41%), and high sugar foods (WTbT: −11.69%, 95%CI: −11.86% to −11.51%). Taxes applied to individuals on high incomes could be higher than if applied to the general population (WTbT: 15.31%, 95%CI: 14.98% to 15.27%). Subsidies were less popular if directed to people living with overweight and obesity (WTbT: −26.43%, 95%CI: −26.80% to −26.07%) or to families with children (WTbT: −15.06%, 95%CI: −15.45% to −14.67%) than to the general population. Subsidies that supported those on low incomes were slightly preferred to subsides provided to the general population (WTbT: 2.24%, 95%CI: 1.89% to 2.58%). Subsidies for fresh produce were slightly preferred to subsidies for high fibre foods (WTbT: 3.01%, 95%CI: 2.83% to 3.20%).

For sustainability taxes, taxing foods whose production generates plastic (WTbT: 29.01%, 95%CI: 22.87% to 35.13%), generates green-house gases (WTbT: 17.60%, 95%CI: 12.18% to 23.02%) or is a meat or dairy product (WTbT: 9.09%, 95%CI: 3.75% to 14.44%) were all preferred to taxes on foods whose production uses a lot of water. Preferences were positive for both hypothecation to reducing the cost of low impact foods (WTbT: 7.48%, 95%CI: 3.41% to 11.55%) and to UK environmental projects (WTbT: 6.47%, 95%CI: 1.58% to 11.36%), relative to general taxation. The least preferred policy attribute was providing tax receipts to countries suffering the impacts of climate change (WTbT: −54.73%, 95%CI: −65.12 to −4.33%).

### 3.2 Subsidy reminder behavioural priming

Results from our experiment-within-experiment were null: the coefficient on the interaction of the preference for subsidies and the individual being in the behavioural priming arm of the randomization, subsidy_asc_prime, was 0.11 with a t-ratio (versus 0) of 1.37 (Appendix B). That is, we did not find evidence of the impact of reminding respondents that subsidies are taxpayer-funded impacted on the experimental choices.

### 3.3 Demographics, attitudes, and diets shape preferences for fiscal policies

**Fig. 2:**
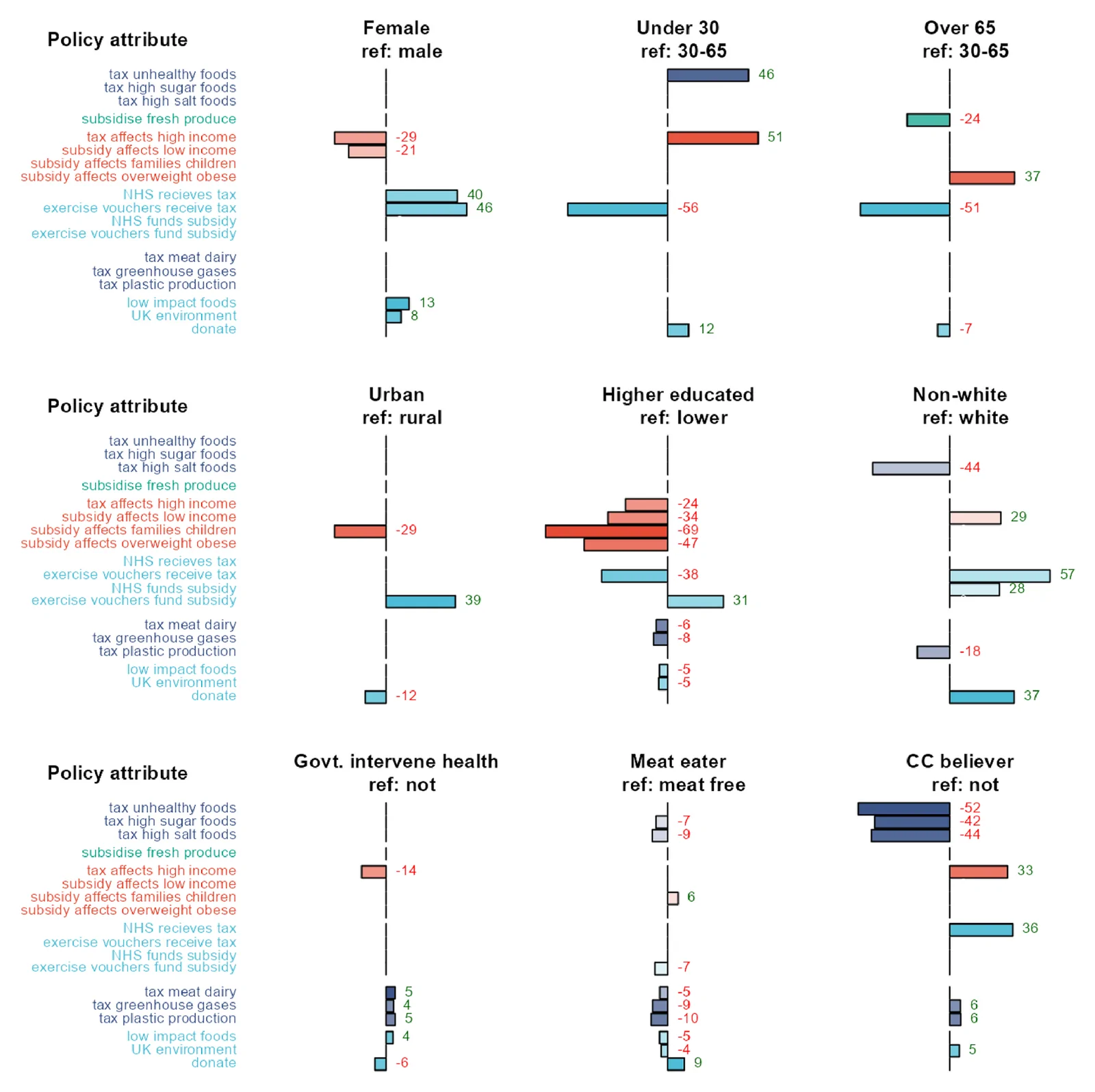
Preference variation for fiscal policy attributes by demographics, diets, and attitudes. Since these are interactions, they are additive to the base. For example, the sum for non-white would be base + interaction, which is -55 + 37 = -18. So still negative, but less so than for white respondents (who are the base, -55).

Fig. 2 shows how preferences vary across demographics, behaviours, and attitudes.

In preliminary analyses, we tested for heterogeneity across regions and countries within the UK. We did not find any evidence for heterogeneity. As a result, and to restrict the dimensionality of the model (9 regions and 3 non-English countries – hence 11 interaction terms for each attribute level), we excluded this from the analysis.

The top two rows of Fig. 2 show preference variation by demographics.

The top row shows that those under 30 had positive preferences for taxing unhealthy foods (WTbT: −46%), for taxing individuals on high incomes (WTbT: 51%), and for donating tax proceeds to countries affected most by climate change (WTbT: 12%). Those under 30 (WTbT: −56%) and those over 65 (WTbT: −51%) had large, negative preferences for subsidised exercise vouchers. In other words, those between 30 and 65 were much more welcoming of subsidised exercise vouchers than their younger or older counterparts. Those over 65 disliked subsidies for fresh produce (WTbT: −24%) and for donating tax revenues to countries affected most by climate change (WTbT: −7%) but were supportive of subsidising foods for those living with overweight and obesity (WTbT: −37%). Female respondents had lower preferences for taxes being applied to those on high incomes (WTbT: −29%) and for subsidies supporting families on low incomes (WTbT: −21%) compared with male respondents. Female respondents had stronger preferences for the NHS receiving hypothecated taxation (WTbT: 40%), for taxes being used to subsidise exercise vouchers, (WTbT: 46%), for taxation revenue supporting subsidies for low environmental impact foods (WTbT: 13%) and for UK environmental projects (WTbT: 8%) compared with male respondents.

The middle row of Fig. 2 shows those from non-white ethnic groups preferred least to tax high salt foods (WTbT: −44%) and plastic production (WTbT: −18%). Preferences on this group were positive for subsidising foods for those on low incomes (WTbT: 29%), subsidies being raised from NHS budgets (WTbT: 28%), providing exercise vouchers (WTbT: 57%), and for donating to countries who suffer negative consequences of climate change (WTbT: 37%). Preferences of those having undergone higher education (versus those not having done so) were large and negative for subsidising foods for families with children (WTbT: 69%), those living with overweight and obesity (WTbT: 47%), and for subsidising exercise vouchers (WTbT: −38%). Further, this group had negative preferences for taxing individuals on high incomes (WTbT: −24%) and for subsidising foods for people on low incomes (WTbT: −34%). The latter might be attributable to the association of university education and higher income (in our data: average income with university education is £45,101 and without is £28,850). In both of these settings, policies of this nature would be more popular amongst those who have not undergone higher education. Those in urban settings (versus those in rural settings) did not support subsidising foods for people on low incomes (WTbT: −29%), nor donating tax revenues (WTbT: −12%). They did support exercise vouchers being used to fund subsidies (WTbT: 39%).

The bottom row shows preference variation by diets and attitudes. For example, the bottom right panel shows that those who believe in climate change had much stronger preferences for taxing sugary drinks than for those who do not believe in climate change (and those who believe less in climate change prefer to tax foods over sugary drinks). WTbT for taxing unhealthy foods, high sugar foods, and high salt foods is lower (WTbT: −52%, −42%, −44% respectively) than for the reference category of taxing sugary drinks. Climate change believers had stronger preferences for taxing individuals on high incomes (WTbT: 33%) and for tax proceeds being hypothecated to the NHS (WTbT: 36%) than those who do not believe in climate change.

### 3.4 Public ranking of fiscal policies

**Fig. 3:**
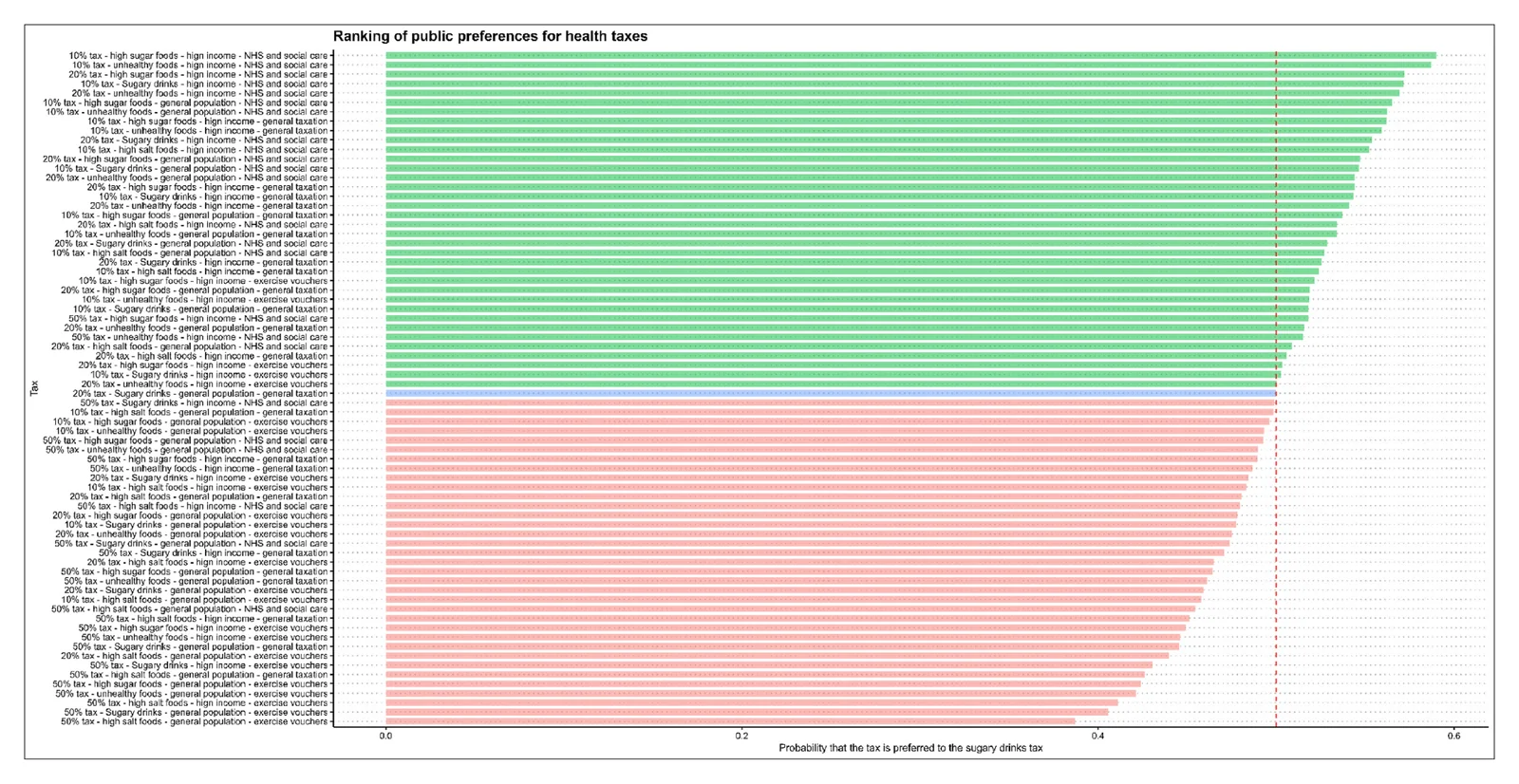
Ranking of taxes in the health DCE. Each observation gives the predicted probability that the policy at hand is preferred to the sugary drinks tax, SDIL, in the UK. Green denotes the policy is preferred to the SDIL; red denotes the SDIL is preferred to the policy. (NB - Our original graph included confidence intervals. However, the graph was difficult to read. We removed them since our interest is in the ranking of these policies. This is available on request.). (For interpretation of the references to colour in this figure legend, the reader is referred to the Web version of this article.)

Fig. 3 shows the preference ranking for health-based taxes that respondents saw in the experiment. The ranking is based on the probability that the tax under study is preferred to the reference, the SDIL tax. Taxes that are more popular than the SDIL include those hypothecated towards the NHS, set at a lower rate, and applied to those on higher incomes. The most popular tax had these characteristics and was applied to high sugar foods. Taxes that are less popular than the SDIL are those whose revenues fund exercise vouchers, are set at a higher rate, and are applied to the general public. The least popular tax had these characteristics and was applied to high salt foods.

**Fig. 4:**
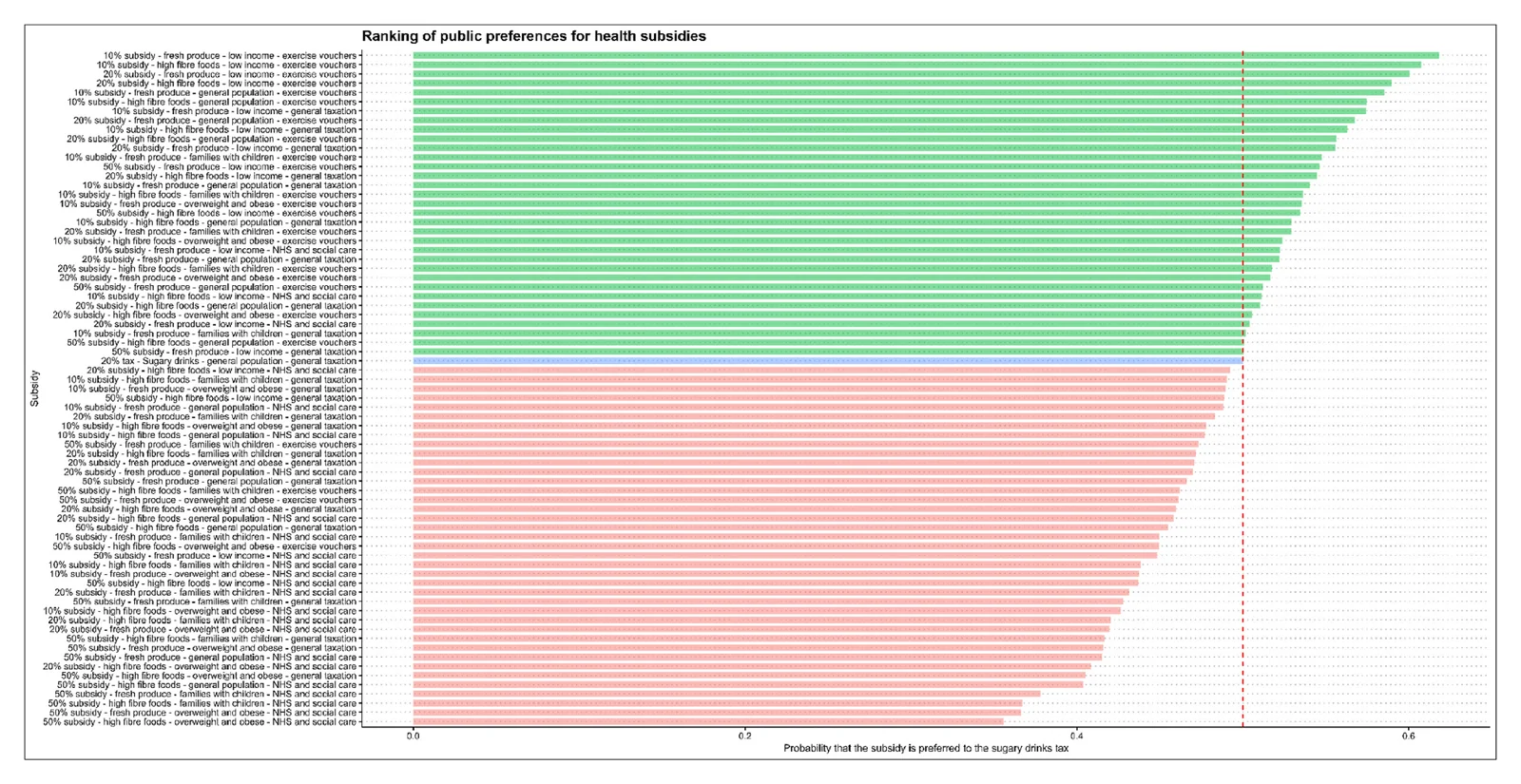
Ranking of subsidies in the health DCE. Each observation gives the predicted probability that the policy and hand is preferred to the sugary drinks tax, SDIL, in the UK. Green denotes the policy is preferred to the SDIL; red denotes the SDIL is preferred to the policy. (NB - Our original graph included confidence intervals. However, the graph was difficult to read. We removed them since our interest is in the ranking of these policies. This is available on request.). (For interpretation of the references to colour in this figure legend, the reader is referred to the Web version of this article.)

Fig. 4 shows the preference ranking for health-based subsidies that respondents saw in the experiment. As above, the ranking is based on the probability that the subsidy at hand is preferred to the reference, the SDIL tax. Subsidies that are more popular than the SDIL include thos that are funded by taking money away from exercise vouchers, are set at a lower rate, and support families on low incomes. The most popular subsidy had these characteristics and was applied to fresh produce. Subsidies that are less popular than the SDIL are those that are funded by the NHS budget, are set at a higher rate, and support families with children or individuals living with overweight and obesity. The least popular subsidy had these characteristics and was applied to high fibre foods.

**Fig. 5:**
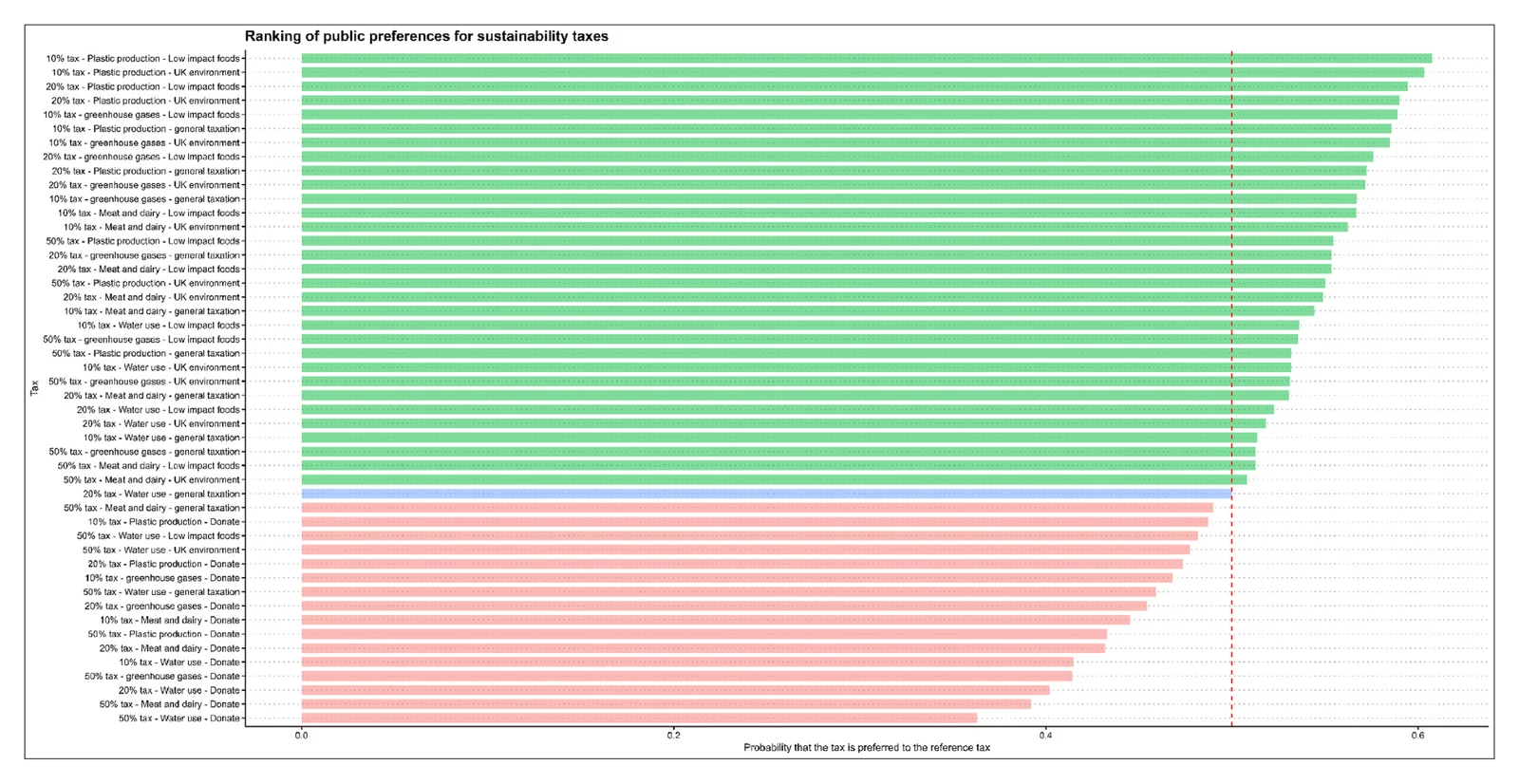
Ranking of taxes in the sustainability DCE. Each observation gives the predicted probability that the policy and hand is preferred to the reference tax of a tax of 20% applied to meat and dairy the proceeds of which go towards general taxation. Green denotes the tax is preferred to the reference tax; red denotes the reference tax is preferred to the tax. (NB - Our original graph included confidence intervals. However, the graph was difficult to read. We removed them since our interest is in the ranking of these policies. This is available on request.). (For interpretation of the references to colour in this figure legend, the reader is referred to the Web version of this article.)

**Fig. 6:**
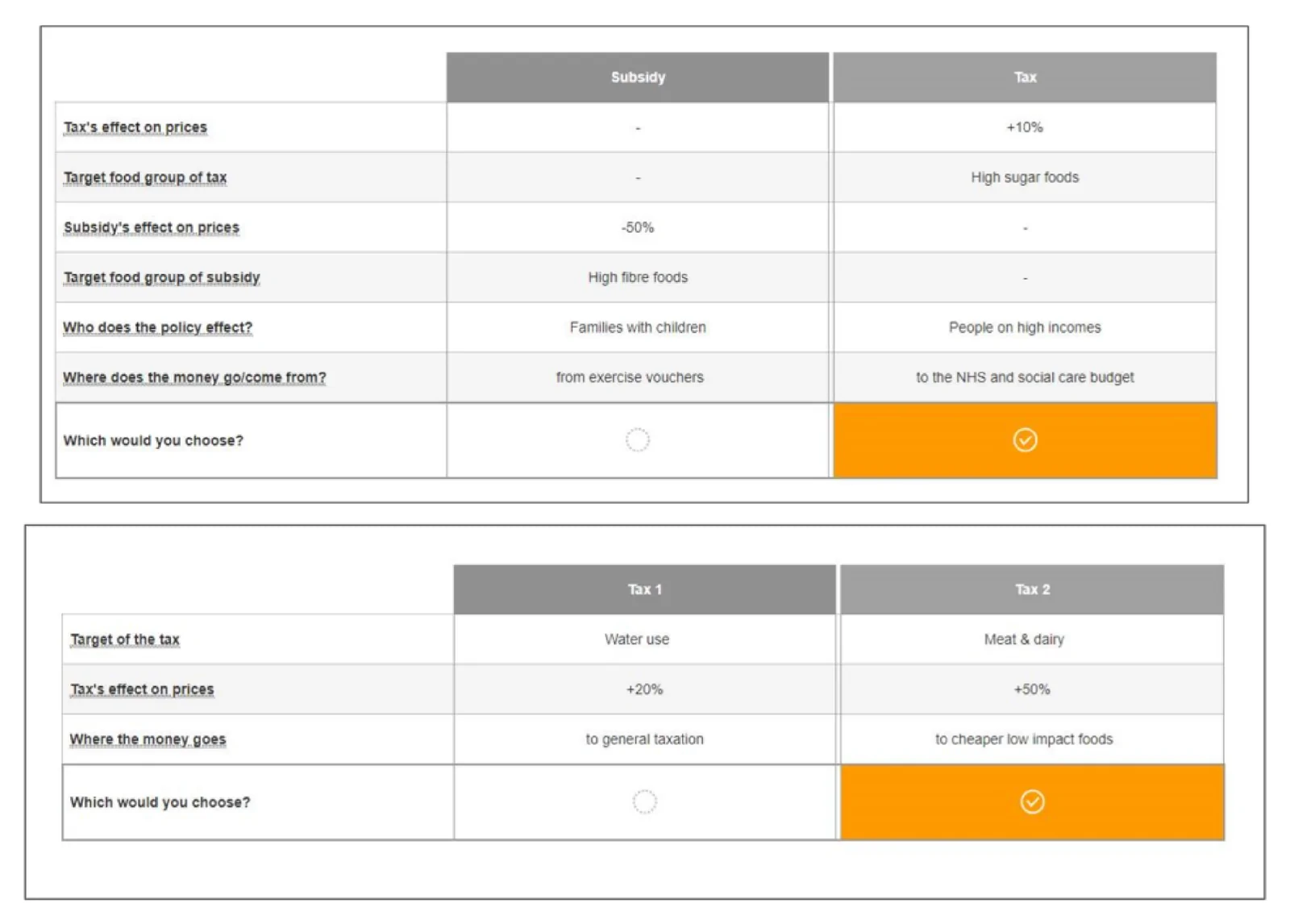
Experimental choice tasks. Above: health DCE choice task, below: sustainability DCE choice task.

**Fig. 7:**
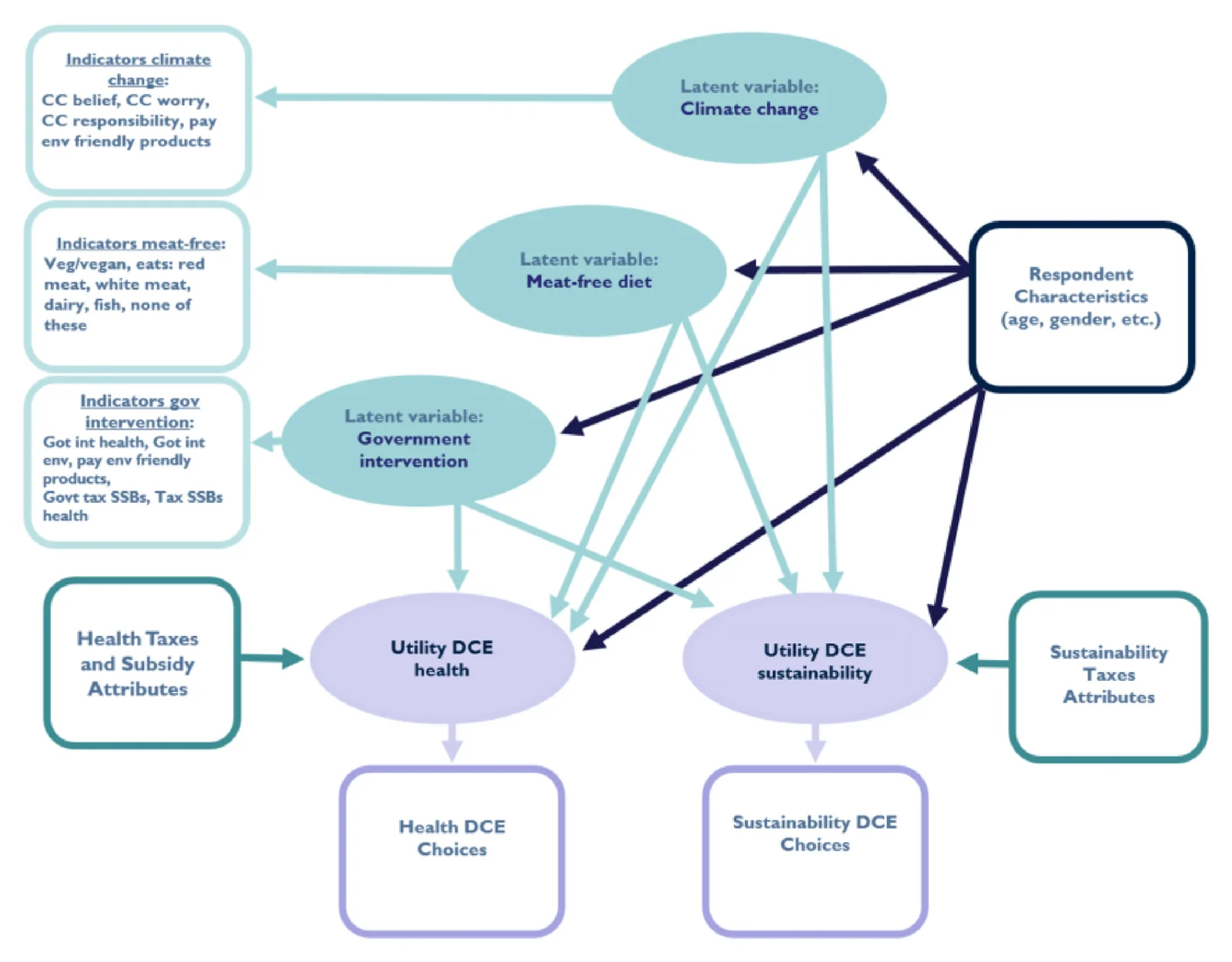
System of equations. Boxes are observed data; ellipses are latent variables. Objects with arrows pointing away from them signify that the object is treated as an independent variable; objects with arrows pointed towards them signify that the object is treated as a dependent variables. Indicator variables (light green boxes) are explained by latent variables (light green ellipses). Latent variables are explained by respondents’ characteristics (dark blue box). Utility (purple ellipses) is explained by attributes and levels (dark green boxes), respondents’ characteristics, and latent variables. Experimental choices (purple boxes) are explained by utility. (For interpretation of the references to colour in this figure legend, the reader is referred to the Web version of this article.)

Fig. 5 shows the preference ranking for sustainability-based taxes that respondents saw in the experiment. The ranking is based on the probability that the policy at hand is preferred to the reference of a 20% tax on foods whose production is high in water use and whose revenues are allocated to general taxation. Taxes that are more popular than the reference include those that are set at a lower rate, applied to foods with high plastic production, and whose revenues are used to support low environmental impact foods. Taxes that are less popular than the reference include those that are set at a higher rate and whose revenues are donated to countries who suffer the effects of climate change.

The range of the forecasts was comparable between health (most popular policy probability: 0.62 – least popular policy probability: 0.36 = 0.26) and sustainability (most popular policy probability: 0.61 – least popular policy probability: 0.36= 0.25). Thus, amongst these policies, there was no outright preferred area for policies to be implemented.

## 4 Discussion

We estimated public support in the UK population for food-based fiscal policies and their attributes using two discrete choice experiments. Policy effectiveness is being measured indirectly by the impact on prices and not on the resulting behavioural response. Among the health policies explored, protecting/enhancing the NHS funding was prioritised most. Individuals were willing to be taxed 22% more if revenues were hypothecated to the NHS funding rather than to general taxation; subsidies should be 25% lower if raised from the NHS budget than from general taxation for individuals to be equally as content with the policy. For sustainability-based policies, donating tax revenues to countries that suffer the impacts of climate change was the least preferred policy attribute. Here, individuals would prefer to be taxed 55% more if the proceeds went to general taxation than if proceeds went to countries that suffer the impacts of climate change. We found substantial variation across gender, age, ethnicity, education, diets, and climate change belief. We ranked 192 policies in order of public preference. The overall order of magnitude of public support for the health-based policies used in this experiment was the same as for the sustainability-based policies.

Similarly to Biondi et al. (2019), we find evidence of response asymmetry between the levels of taxes and subsidies (significant parameter on level of tax but not for level of subsidy), though we did not find evidence of difference in preference for a subsidy over a tax (non-significant parameter of subsidy vs. tax), nor did we find evidence that reminding individuals that subsidies are paid using public funds impacted policy preferences. This could be because taxes applied to products can be avoided (by avoiding purchasing taxed products), whereas subsidies, funded by taxes, cannot easily be. That attitudes and perceptions dictate public opinions on sustainability policies corroborates the findings of other studies (Mazzocchi et al., 2015; Dieteren et al., 2022; Bergquist et al., 2022). More specifically, the favourability of progressive taxes that we found is in keeping with the finding that fairer taxes have previously garnered more public support (Bergquist et al., 2022). The demographic variation in our policy support results do not align with Pechey et al. (2022), who did not find policy acceptability differences by demographics. However, this difference might be explained by our study design and analysis being specifically designed to assess nuance variation across demographic groups. Our results do align with those of Pechey et al. in showing that attitudes to climate change and the government, as well as food behaviours, relate to variation in policy preferences.

Our experimental design drew on multiple sources that included a restricted review of global food-based fiscal policies and regular input from subject experts and food industry representatives. Defining the attributes and levels of policies using a scoping review of existing literature ensured we captured preferences for a comprehensive array of feasible policy options. Co-production with Patient and Public Involvement (PPI) members of the project team resulted in, for example, the use of an additional latent variable related to meat-free preferences. This ensured the most policy-relevant content in the experimental design but also, importantly, that the experiment was accessible and easily understood by those who responded. In addition, we exploited the fact that a sugary drinks policy was already in place in the UK by using this in the experiment. In this way, we sought to increase the realism of the experiment and enhance the data quality (Train and Wilson, 2009). We note, however, that it was not feasible to include the specific details of the SDIL (i.e. that it is a tiered levy applied to producers according to the sugar content of drinks), as we needed policies to be easily understood by participants. Instead, we used a general tax on “sugary drinks” whose level could vary (i.e. could be 10%, 20%, or 50%) reflecting that the SDIL itself varies. Lastly, we used an experiment-within-experiment design to test the impact of a reminder of financial recourse for subsidies on the preferences for subsidies (though the result was null). We did not specify the source of the subsidy (e.g. VAT, income tax, etc.), which may have impacted choices. However, to do so would have added additional complexity to the experiment and the interpretation of the data. This may be explored in future research. We had a large, national sample of the population in all four UK countries. This allowed for a rich analysis of heterogeneity. Indeed, it is innovative to apply the modelling approach in a health context, and it allowed for joint modelling of the choice responses and the responses to attitudinal questions, a rich heterogeneity analysis, and an appropriate comparison of effect sizes between experiments. We applied statistical diagnostics to ensure that our models were robust and captured important intricacies in respondents’ choices.

Limitations include that stated choice studies are subject to hypothetical bias i.e. people’s stated behaviour in surveys may differ from their behaviours in real world settings (Buckell et al., 2020). However, in this setting, this is of less concern than for other studies because we are interested in public support for policies, which itself is stated preference (though we recognise there remains scope for individuals to report untruthfully, e.g. social desirability (Nederhof, 1985) or with obfuscation (Chorus et al., 2021)). We asked respondents, “We would like you to look at the features of each tax and subsidy and choose which of them you think is better for public health”. We assumed that individuals made choices as they would if they were actually voting, but they may have had alternative ways of answering (self-interest, for example) that are not observed by us. Further, stated preference studies are prone to overestimating willingness-to-pay (Buckell and Hess, 2019), hence our WTbT measures in absolute value may have been overstated. Our focus here is relative, rather than absolute, preference. Relatedly, it is hard to know if respondents understood the fiscal implications of the 10%/20%/50% levels of the taxes/subsidies presented. In our analysis, however, we used the level of tax as a denominator to express preferences in tax % terms and compare preferences across the two experiments. Thus, the impact of this issue is diminished. We did not distinguish between personal and household income. It may be the case that personal income we recorded here was highly correlated with the household income; in other cases, less so (in reality, of course, we simply do not know). It is not clear if respondents, when responding to the tasks, had in their mind their personal or household income. We are limited insofar as we are only able to observe correlations between choices and reported personal income. We used a policy that has recently been introduced in our design, the SDIL, aiming to anchor participants’ thinking and further reduce hypothetical bias. We acknowledge an issue in that meat-related consumption which was used in the latent variable of meat-free diets may not correspond to unhealthy diets as in the experiment. While imperfect, we believe that this may correlate with some of the foods. For example, “fresh produce” pertains to fruit and vegetables, which would correlate very strongly with meat-free diets (and strongly, negatively with meat-heavy diets). Finally, we instructed respondents that their responses would be used for a scientific study, which has been shown to reduce hypothetical bias (Wuepper et al., 2019). An arguable limitation is the lack of a “no policy”, opt-out option in the experiment. However, in this study we are interested in relative policy preferences rather than whether to implement a given policy per se over doing nothing. Nonetheless, this design feature may have resulted in upwardly biased measures on WTP in ways that are not observed to us in our data. There is prior evidence suggesting that WTP can be inflated in stated choice tasks (Wuepper et al., 2019). We note that policy preferences may evolve over time and this paper measures a static cross-section of preferences for policies. Our measure of government trust was specific to the food policy setting in which we asked survey questions and in which the survey was based. Lastly, the design limited the number of policies for which we could simulate preferences. However, preferences for 192 policies were simulated, which is far greater than if we had used alternative study designs. Our findings are limited to the UK context. However, our approach is not. Researchers in other settings can adopt our approach of using multiple experiments in adjacent policy areas (i.e. health and sustainability) within the same sample and modelling those data to enable comparisons that can inform multiple policies. Our code is shared with this intention.

The need for food system reform in the UK for health and environmental benefit has been set out by various authoritative bodies, and fiscal policies have been proposed to address such issues (UK Health Alliance of Climate Change, 2020; NFS, 2021 ; Liberal Democrats, 2024).

There was no clear preference for health- or sustainability-based food fiscal policies. Thus, maximising public support depends on the features of a particular policy, rather than if it is health-based or sustainability-based. This finding could help inform the focus/strategy of advocacy efforts (e.g., food-related NGOs) who are actively working to pursue the implementation of further food-related fiscal policies.

The most popular health-based policies overall were those which protected the NHS budget and taxes that were directed towards those on high incomes. The former were preferred more by women and less by those from non-white ethnic groups. Whilst evidence shows hypothecation is popular in other policy contexts, it can be difficult to achieve in practice. It is argued by some to be a convenient fiction, and the UK government have explained that the collection and spending of tax revenues are separate pursuits (Select Committee, 2008). The latter were preferred by those under 30 and those with a strong belief in climate change; and preferred less by female respondents, those with higher education, and those who believe the government should intervene in food markets. Operationalising taxes on higher income earners is nontrivial. Means-testing, popularised in the UK for issuing benefits, would be feasible in the food purchasing setting, as per the case for NHS Health Start vouchers subsidising healthy foods. Adding additional taxes to items purchased disproportionately by those on high incomes is feasible, e.g. the higher quality ranges offered by supermarkets, but undermined if those items are avoided. In the absence of effective implementation mechanisms, this policy option may be redundant. In any case, the marginal rate of tax falls as incomes rise, degrading the effect of progressive taxation. Note, however, that these issues, as in the case for NHS hypothecation, pertain to implementation rather than to popularity, which is the interest here.

The most popular sustainability-based policies overall were those applied to foods with high plastic production and greenhouse gas emissions. These policies were preferred slightly more by those who believe governments should intervene in markets and by those with strong climate change beliefs. That these policies were preferred to taxes on water-intensive foods may be explained by the context sample included in our study; repeating our study in a country/state whose food system is vulnerable to the impact of droughts/water shortages may find different results. These policies would be straightforward to impose, but other practical considerations would be needed. For example, if foods purchased more by people on low incomes had high plastic production, they would be taxed more. In this case, the tax would in effect be regressive.

Across both experiments, the least popular policies were those donating tax revenues to countries who suffer the effects of climate change. These policies were disliked to a lesser degree by those under 30, those from non-white ethnic groups, and high meat eaters. However, preferences in these groups were still negative. That is, this policy feature was disliked at the sample level and by all 9 subgroups whose preferences were measured. These policies would not be well-supported if advocated by policymakers.

## Supplementary Material

Supporting Information

## Figures and Tables

**Table 1:** Attributes and levels used in the discrete choice experiments. The attributes are listed on the far left, and the levels each attribute could take, and for which alternative, are in the columns in the middle and to the right, which show the alternatives for those levels, e.g. “Fresh produce” can only occur for subsidies, but “50%” could occur for a tax or subsidy). Descriptions of these used in the survey are presented in Appendix A. Combinations of attribute levels make up the choice tasks. A sample choice task is shown in Fig. 6. (ref.) denotes the reference level for each attribute in analyses.

Panel A: Health DCE design				
		Tax		Subsidy
**Effect on prices**		10%		–10%
		20%		– 20%
		50%		– 50%
**Target foods**				Fresh produce
				High fibre foods (ref.)
		Sugary Drinks (ref.)		
		Unhealthy foods		
		High sugar foods		
		High salt foods		
**Who the policy affects**		General population (ref.)		General population (ref.)
				People on low incomes Families with Children
		People on high incomes		
				People living with overweight and obesity
**Where the money goes/comes from**		General taxation (ref.)		General taxation (ref.)
		NHS & social care		NHS & social care
		Exercise vouchers		Exercise vouchers
**Panel B: Sustainability DCE design**				
		**Tax 1**		**Tax 2**
**Increase in prices**		10%		10%
		20%		20%
		50%		50%
**Target of the tax**		Water Use (ref.)		Water Use (ref.)
		Greenhouse gases		Greenhouse gases
		Plastic production		Plastic production
		Meat & Dairy		Meat & Dairy
**Where the money goes**		Cheaper low impact foods		Cheaper low impact foods
		Government spending on UK green projects		Government spending on UK green projects
		Donations to countries that are harmed by climate change		Donations to countries that are harmed by climate change
		General taxation (ref.)		General taxation (ref.)

## Data Availability

Analytical code is available on github here: https://github.com/johnbuckell/COPPER-project-DCE-model.
