# Supplementary material for: Characterising public opinion for food-based fiscal policies in the UK
Source: Soc Sci Med. Author manuscript; Available in PMC 2026 Oct 19. (PMC7619597; doi:10.1016/j.socscimed.2026.119269)
Supplement: Supplementary file 1 [file EMS218750-supplement-Supporting_Information.docx]

Appendices for “Characterising public opinion for food-based fiscal policies in the UK”

Contents

[Appendix A: Description of attributes in the survey	1](#_Toc171404445)

[Example of choice tasks	20](#_Toc171404446)

[System of equations figure	21](#_Toc171404447)

[Appendix B: Full model results	22](#_Toc171404448)

[Appendix C: Weighted versus non-weighted model	31](#_Toc171404449)

[Appendix D: Data quality supplemental statistics	33](#_Toc171404450)

[Appendix E: Descriptive statistics for sample and indicator variables used in the latent variables	34](#_Toc171404451)

[Appendix F: Rationale for the use of latent variables	36](#_Toc171404452)

[Appendix G: Description of scoping review methods	37](#_Toc171404453)

[Appendix H: Full literature search strategy	44](#_Toc171404454)

## Appendix A: Description of attributes in the survey

COPPER Survey: Preferences for health and environmental policies

**//////////////////**

**Start of Block: Section 1A: Screening/quota Questions**

**//////////////////**

*This survey is part of a scientific study led by the University of Oxford which aims to help the government decide which policies are best to help improve people’s health and to help the environment. Your answers will provide very useful information for this important research study. The results may help the government to make the best decisions that they can. Therefore, please answer the questions as carefully and truthfully as you can. Thank you.*

It helps us to know some basic information about the people who take part in this survey. Please tell us about yourself in the following questions.

1 **Which region do you currently live in?**

o North East

o North West

o Yorkshire & The Humber

o East Midlands

o West Midlands

o East of England

o London

o South East

o South West

o Scotland

o Wales

o Northern Ireland

2 **How old are you?**

**[drop-down box 0-110]**

3 **What is your gender?**

o Male

o Female

o Non-binary

o Prefer not to say [-> eject from survey]

**//////////////////////////////////**

End of Block: Section 1A: Preliminary Questions

**//////////////////////////////////**

**////////////////////////////////**

Start of Block: Section 1B: Preliminary Questions

**////////////////////////////////**

4 **Which of these qualifications do you have?**

[ ] Higher education: Degree or higher

[ ] Secondary education: A levels

[ ] Other vocational/work-related qualifications

[ ] None of the above qualifications

End of Block: Section 1B: Preliminary Questions

5 **What is your ethnicity? (Please select all that apply.)**

Asian or Asian British

o	Indian

o	Pakistani

o	Bangladeshi

o	Chinese

o	Any other Asian background

Black, Black British, Caribbean or African

o	Caribbean

o	African

o	Any other Black, Black British, or Caribbean background

Mixed or multiple ethnic groups

o	White and Black Caribbean

o	White and Black African

o	White and Asian

o	Any other Mixed or multiple ethnic background

White

o	English, Welsh, Scottish, Northern Irish or British

o	Irish

o	Gypsy or Irish Traveller

o	Roma

o	Any other White background

Other ethnic group

o	Arab

o	Any other ethnic group

6 **Currently, do you live in an area that is:**

o Rural

o Suburban

o Urban

**////////////////////////////////**

End of Block: Section 1B: Preliminary Questions

**////////////////////////////////**

**////////////////////////////////**

Start of Block: Randomization Prime

**////////////////////////////////**

Subsidies reduce the cost of things people want to buy and can be used, for example, to make healthier foods cheaper/ more attractive.

Government subsidies are paid for from general taxation, which is collected from all UK tax payers. This means that, If you pay taxes, you also pay for subsidies.

**////////////////////////////////**

End of Block: Randomization Prime

**////////////////////////////////**

**////////////////////////////////**

Start of Block: DCE and follow-up questions

**////////////////////////////////**

#####################################

DCE 1: food and nutrient taxes and subsidies

#####################################

A food tax is money added to the price of a food or a drink, which is put in place by the government. Taxes vary in size and can be set for several reasons, for example to put people off buying unhealthy foods or to raise money to spend on public services, such as the NHS or the police force.

A food subsidy is a decrease in the price of a food or a drink, put in place by the government. Subsidies can be set for several reasons, for example to encourage people to buy healthier foods.

We are giving you six tasks, which show different kinds of food taxes and subsidies that could be introduced by the government. These taxes and subsidies are applied to foods and are **aimed at helping people make healthier food choices**.

** Please note that the items and wording used here are taken from existing sources to try to make choices looks and sound realistic. They are not meant to be judgemental. We are only interested in what you think about the taxes and subsidies described by the words. **

In each task, there will be one tax and one subsidy.

The taxes and subsidies have different features, which are listed below.

We would like you to look at the features of each tax and subsidy and choose which of them you think is better for public health. In each task, you will choose between taxes and subsidies that have different features.

The features are:

1. **Effect on price**: this is the amount the price of food will change for each tax or subsidy. The options for this feature will be that prices go up for taxes, or down for subsidies by the following amounts
   - **10%** (prices go up for taxes, and go down for subsidies)

To show you what this means, a 10% tax on a food item that costs £1.00 would change the price to £1.10; and a 10% subsidy on a food item that costs £1.00 would change the price to £0.90.

- **20%** (prices go up for taxes, and go down for subsidies)

To show you what this means, a 20% tax on a food item that costs £1.00 would change the price to £1.20; and a 20% subsidy on a food item that costs £1.00 would change the price to £0.80.

- **50%** (prices go up for taxes, and go down for subsidies)

To show you what this means, a 50% tax on a food item that costs £1.00 would change the price to £1.50; and a 50% subsidy on a food item that costs £1.00 would change the price to £0.50.

2. **Target foods**: this will be the specific types of foods that the taxes or subsidies will be applied to the tax or subsidy will only affect prices for these foods.

The options for this feature will be:

- **Unhealthy foods** (any food that has high levels of fat, sugar, or salt)
- **Fresh produce** (i.e. fruit and vegetables)
- **High fibre foods** (e.g. grains, wholemeal bread)
- **High sugar foods** (the more sugar in a food, the higher the tax)
- **High salt foods** (the more salt in a food, the higher the tax)
- **Sugary drinks** (fizzy drinks that are high in sugar, e.g. cola, lemonade)

3. **Who the policy affects**: this refers to the groups of people who will be affected by the tax or subsidy of the tax or subsidy. The options for this feature will be:
   - **Families with children**
   - **General population** (everybody – no targeted group)
   - **People on low incomes** (less than £17,000 per year)
   - **People on high incomes** (more than £50,000 per year)
   - **People who are with overweight and obesity**
4. **Where the money goes/comes from**: this where the money that is collected by taxes goes, or where the money comes from to pay for subsidies.

The options for this feature will be:

- **Exercise vouchers** (e.g. free gym or swimming pool passes, as per the NHS’s GP Exercise Referral scheme)

For taxes, this means that the tax on foods collected by this tax would be used to pay for exercise vouchers, like those provided by GPs to help people lose weight

For subsidies, this means that money that was going to be spent on exercise vouchers to help people lose weight is instead used to pay for the food subsidy.

- **The NHS and social care budget**

For taxes, this means that the tax on foods collected by this tax would be put towards the NHS budget to help pay for all of the services that the NHS provides.

For subsidies, this means that money would be taken out of the NHS budget and is instead used to pay for the food subsidy.

- **General taxation** (could be spent on anything by the government)

For taxes, this means that the tax on foods collected by this tax would go to the government to help pay for all of the services for anything that the government provides (education, NHS, law and order, etc.).

For subsidies, this means that money would be taken out of the government’s total budget and is used to pay for the food subsidy rather than services that the government provide.

We will now ask you to make the six choices between taxes and subsidies. Please think about these features when making your choices.

Each of the tasks we show you will have different combinations of the 4 features listed above

1. **Effect on price**
2. **Target foods**
3. **Who the policy affects**
4. **Were the money goes/comes from**

We would like you to choose your favourite tax or subsidy from the available options. This choice is repeated 6 times, with different features each time.

You might make different choices as the tax and subsidy options change. This is fine, what is important is to choose what seems the better choice for you on each time – even if neither feels quite right for you.

The graphic below gives an example choice like those that you will be asked to answer. Please pick the one that you would most prefer.

**You have indicated that you would choose XXX. Is this the option you meant to select?**

o Yes, this is correct

o No, this is not correct

Display This Question:

If you have indicated that you would choose = No, this is not correct

**Please try again.**

Display This Question:

If you have indicated that you would choose = Yes, this is not correct

[insert warm up task here]

Now the real choices! Please make 6 choices, as you did on the previous page. The features of each tax and subsidy will be different in each question. Your answers will provide very useful information for a research study to help us understand the kinds of taxes and subsidies that people most want to help people make healthier choices.

#####################################

DCE 2: environmental taxes for foods

#####################################

As a reminder, food taxes are increases in the price of a food or a drink put in place by the government. Taxes vary in size and can be set for several reasons, for example to put people off buying unhealthy foods or to raise money to spend on public services, such as the NHS or the police force.

As a reminder, a food subsidy is a decrease in the price of a food or a drink, put in place by the government. Subsidies can be set for several reasons, for example to encourage people to buy healthier foods.

We are giving you six tasks, which show different kinds of food taxes that could be introduced by the government. These taxes are applied to foods and **are aimed at reducing the amount of foods people eat that harm the environment more than others**.

** Please note that the items and wording used here are taken from existing sources to try to make choices looks and sound realistic. They are not meant to be judgemental. We are only interested in what you think about the taxes described by the words. **

In each task, there will be two taxes. The taxes will be defined by a set of features. We would like you to look at the features of each tax and choose which of them you think is better. The features will change in each tasks, showing different taxes for you to choose between each time. The features are below:

1. **Increase in price**: this is the amount the food price will change for each tax. The options for this feature will be:
   - **10%**, the price of a food that harms the environment will increase by 10%

To show you what this means, a 10% tax on a food item that costs £1.00 would change the price to £1.10

- **20%**, the price of a food that harms the environment will increase by 20%

To show you what this means, a 20% tax on a food item that costs £1.00 would change the price to £1.20

- **50%**, the price of a food that harms the environment will increase by 50%

To show you what this means, a 50% tax on a food item that costs £1.00 would change the price to £1.50

2. **Target of the tax**: this will be the types of environmental harm that the taxes will be applied to. That is, the tax affects prices for foods that produce more of these types of harms. The options for this feature will be:
   - **Water Use**, foods that are produced using high amounts of water are taxed
   - **Greenhouse gases**, foods that cause high amounts of greenhouse gases to be emitted in their production are taxed
   - **Plastic production**, foods that are made using high amounts of plastic (e.g. with lots of plastic packaging) are taxed
   - **Meat and dairy**, which generally cause more harm to the environment in their production than other foods are taxed
3. **Where the money goes**: this is the place where the money that is collected goes. The options for this feature will be:
   - **Vouchers for environmentally friendly foods** (e.g. vegetables grown in the UK)
   - **Government spending on green projects** (e.g. to fund new wind farms for the UK)
   - **Donations to countries that are harmed by climate change** (e.g. Pacific Islands that are vulnerable to flooding because of climate change)
   - **General taxation** (could be spent on anything by the government)

We will now ask you to make the six choices between different taxes. Please consider these features when making your choices.

We will show you each choice task with different combinations of the 3 features just described:

1. **Increase in price**
2. **Target of the tax**
3. **Where the money goes**

We will ask you to choose your preferred tax from the available options. This choice is repeated 6 times, with different features each time.

You might pick different choices as the tax options change. What is important is to choose what seems to be the better choice for you on each occasion – even if neither feel quite right for you.

Just before you answer 6 choices, there is a warm-up question to help you see how the choices work. The graphic below gives an example choice like those that you will be asked to answer. Please pick the one that you would most prefer.

**You have indicated that you would choose XXX. Is this the option you meant to select?**

o Yes, this is correct

o No, this is not correct

Display This Question:

If you have indicated that you would choose = No, this is not correct

**Please try again.**

Display This Question:

If you have indicated that you would choose = Yes, this is not correct

Now the real choices! Please make 6 choices, as you did on the previous page. The features of each tax will be different in each question. Your answers will provide very useful information for a research study to help us understand the kinds of taxes that people most want to help protect the environment.

21 **To what extent do you agree with the following statements?**

|  | Strongly disagree (1) | Disagree  (2) | Somewhat disagree  (3) | Neither agree nor disagree (4) | Somewhat Agree  (5) | Agree  (6) | Strongly agree (7) |
| --- | --- | --- | --- | --- | --- | --- | --- |
| Taxes are worse than subsidies (1) | o | o | o | o | o | o | o |
| It is better to tax fat than salt (2) | o | o | o | o | o | o | o |
| We should aim taxes at adults more than children (3) | o | o | o | o | o | o | o |
| Protecting the environment is more important than if people get healthier (4) | o | o | o | o | o | o | o |

/////////////////////////////////////////////

End of Block: DCE and follow-up questions

/////////////////////////////////////////////

/////////////////////////////////////////////

Start of Block: Food Behaviours

/////////////////////////////////////////////

**Would you describe yourself as:**

o Omnivore (eats everything)

o Vegetarian

o Vegan

o Pescatarian

oFlexitarian

o Other, please specify: ________________________________________________

o Prefer not to answer

**Which of the following dairy foods do you eat?**

o Whole milk

o Semi-skimmed milk

o Soya milk

o Other sort of milk

o Don’t drink/use milk

**[If QXX = vegetarian OR vegan, skip to next section] In the past year, have you made efforts to consume more or less plant-based foods like beans, lentils, nuts, seeds?**

o Consume more

o Consume less

o No effort made (about the same)

o Don’t know

o Prefer not to answer

**Which of the following foods do you eat?**

o Dairy (cheese, milk, butter)

o Red meat

o White meat

o Fish

o Other, please specify: ________________________________________________

o Prefer not to answer

**About how much do you spend per week on:**

|  | Nothing | £0-5 | £5-10 | £10-15 | £15-20 | £20-25 | More than £25 |
| --- | --- | --- | --- | --- | --- | --- | --- |
| Beef or Lamb | o | o | o | o | o | o | o |
| Pork, Ham, Bacon | o | o | o | o | o | o | o |
| Poultry | o | o | o | o | o | o | o |
| Fish | o | o | o | o | o | o | o |
| Non-alcoholic drinks | o | o | o | o | o | o | o |

/////////////////////////////////////////////

End of Block: Food Behaviours

/////////////////////////////////////////////

/////////////////////////////////////////////

Start of Block: Food and Environment Attitudes

/////////////////////////////////////////////

**Do you believe the Earth’s climate is changing?**

o Yes

o No

**[If QXX = No, skip this question] How worried are you, if at all, about climate change?**

o Not at all worried

o Not very worried

o Somewhat worried

o Very worried

o Extremely worried

**[If QXX = No, skip this question] To what extent do you feel a personal responsibility to try to prevent climate change from worsening?**

[Slider from 1 to 11]

**I would pay more for environmentally-friendly products**

[Slider from 1 to 5 on agreement]

|  | Strongly disagree | Disagree | Neither agree nor disagree | Agree | Strongly agree |
| --- | --- | --- | --- | --- | --- |
| I am happy to pay more for environmentally-friendly products | o | o | o | o | o |
| The government should intervene to improve people’s health | o | o | o | o | o |
| The government should intervene to improve the environment | o | o | o | o | o |
| The government should tax sugary drinks | o | o | o | o | o |
| The government should tax sugary drinks if the money was spent on healthy foods | o | o | o | o | o |

/////////////////////////////////////////////

End of Block: Food and Environment Attitudes

/////////////////////////////////////////////

/////////////////////////////////////////////

Start of Block: Other Demographics

/////////////////////////////////////////////

8 **Do you have any of the following long-term conditions?**

o Heart disease or stroke

o Diabetes

o High blood pressure

o Asthma or other breathing problems

o Mental health problems including depression or anxiety

o Other, please specify: ________________________________________________

o No health problems

o Prefer not to answer

12 **What is your smoking status?**

o Never smoked

o Ex smoker

o Current smoker

o Prefer not to answer

14 **How often do you exercise on average (e.g. go for a walk or a jog)?**

o Never

o 1-2 days per week

o 3-5 days per week

o 6-7 days per week

o Prefer not to answer

24 **How many people are in your household?**

o 1

o 2

o 3

o 4

o 5

o 6

o 7

o 8

o 9

o 10 or more

o I prefer not to answer

23 **Are you currently employed?**

o Yes

o No

o Prefer not to answer

22 **Last year, that is 2021 what was your family’s total income?**

**That is, include the income from all family members living at home and all sources of income. If you do not know, please provide your best guess. Please select one choice below.**

o £10,000-20,000

o £20,000-30,000

o £30,000-40,000

o £40,000-50,000

o £50,000-60,000

o £60,000-70,000

o £70,000-80,000

o £80,000-90,000

o £90,000-100,000

o £100,000-110,000

o £110,000-120,000

o £120,000-130,000

o £130,000-140,000

o £140,000-150,000

o £150,000 or more

o I prefer not to answer

o I do not know

/////////////////////////////////////////////

End of Block: Other Demographics

/////////////////////////////////////////////

/////////////////////////////////////////////////

Start of Block: Quality

/////////////////////////////////////////////////

25 **How carefully and accurately have you answered questions throughout this survey?**

o Not at all

o Not very

o Somewhat

o Extremely

26 **How difficult did you find this survey was to answer?**

o Not at all

o Not very

o Somewhat

o Extremely

27 **Did you think the survey was:**

o Too long

o Too short

o About the right length

o Not sure

28 **Was the choice making easy to understand?**

o Yes

o No

o Not sure

29 **Did any part of the survey make you feel uneasy or uncomfortable?**

o Yes

o No

o Not sure

o If yes, which part ________________

30 **Would you like to make any other comments (we would be very grateful for any feedback)?**

o Yes

o No

o If yes: ________________

////////////////////////////////////

End of Block: Quality

////////////////////////////////////

### Example of choice tasks

Figure A1: Experimental choice tasks. Above: health DCE choice task, below: sustainability DCE choice task.

### System of equations figure

Fig A2: System of equations. Boxes are observed data; ellipses are latent variables. Objects with arrows pointing away from them signify that the object is treated as an independent variable; objects with arrows pointed towards them signify that the object is treated as a dependent variables. Indicator variables (light green boxes) are explained by latent variables (light green ellipses). Latent variables are explained by respondents’ characteristics (dark blue box). Utility (purple ellipses) is explained by attributes and levels (dark green boxes), respondents’ characteristics, and latent variables. Experimental choices (purple boxes) are explained by utility.

## Appendix B: Full model results

|  | Estimate | Rob.std.err. | Rob.t-ratio(0) |
| --- | --- | --- | --- |
| left_to_right_bias_dce1 | 0 | NA | NA |
| tax_asc | 0 | NA | NA |
| subsidy_asc_midpoint | -0.112553831 | 0.130934276 | -0.85962083 |
| subsidy_asc_range | 1.100711671 | 0.060419993 | 18.21767298 |
| subsidy_asc_prime | 0.113968853 | 0.0831786 | 1.370170378 |
| subsidy_asc_over65 | 0 | NA | NA |
| subsidy_asc_under30 | 0 | NA | NA |
| subsidy_asc_female | 0.232497034 | 0.126947819 | 1.831437799 |
| subsidy_asc_nonwhite | 0 | NA | NA |
| subsidy_asc_heduc | 0 | NA | NA |
| subsidy_asc_urban | 0 | NA | NA |
| subsidy_lambda_cc_belief | 0 | NA | NA |
| subsidy_lambda_meat_free_diet | 0 | NA | NA |
| subsidy_lambda_govt_int_health | -0.358203145 | 0.082724832 | -4.330055899 |
| level_of_tax_dce1_midpoint | -4.678486047 | 0.226541907 | -20.65174655 |
| level_of_tax_dce1_range | 0 | NA | NA |
| lambda_income_tax | -0.231048449 | 0.139061308 | -1.661486229 |
| level_of_tax_dce1_over65 | 0 | NA | NA |
| level_of_tax_dce1_under30 | -0.836335907 | 0.309794376 | -2.699648447 |
| level_of_tax_dce1_female | 0 | NA | NA |
| level_of_tax_dce1_nonwhite | 0 | NA | NA |
| level_of_tax_dce1_heduc | 0 | NA | NA |
| level_of_tax_dce1_urban | 0 | NA | NA |
| level_of_tax_dce1_lambda_cc_belief | 0 | NA | NA |
| level_of_tax_dce1_lambda_meat_free_diet | 0 | NA | NA |
| level_of_tax_dce1_lambda_govt_int_health | 0 | NA | NA |
| level_of_subsidy_midpoint | 0.001945998 | 0.002124932 | 0.915792907 |
| level_of_subsidy_range | 0 | NA | NA |
| level_of_subsidy_over65 | 0 | NA | NA |
| level_of_subsidy_under30 | 0 | NA | NA |
| level_of_subsidy_female | 0 | NA | NA |
| level_of_subsidy_nonwhite | 0 | NA | NA |
| level_of_subsidy_heduc | 0 | NA | NA |
| level_of_subsidy_urban | -0.008637251 | 0.003134305 | -2.755714745 |
| level_of_subsidy_lambda_cc_belief | 0 | NA | NA |
| level_of_subsidy_lambda_meat_free_diet | 0 | NA | NA |
| level_of_subsidy_lambda_govt_int_health | -0.00319503 | 0.002115274 | -1.510456708 |
| tax_sugary_drinks | 0 | NA | NA |
| tax_unhealthy_foods_midpoint | 51.4206782 | 26.0526743 | 1.973719765 |
| tax_unhealthy_foods_range | 0 | NA | NA |
| tax_unhealthy_foods_over65 | 0 | NA | NA |
| tax_unhealthy_foods_under30 | 45.53789687 | 29.12208691 | 1.56368934 |
| tax_unhealthy_foods_female | 0 | NA | NA |
| tax_unhealthy_foods_nonwhite | 0 | NA | NA |
| tax_unhealthy_foods_heduc | 0 | NA | NA |
| tax_unhealthy_foods_urban | 0 | NA | NA |
| tax_unhealthy_foods_lambda_cc_belief | -44.0413066 | 16.77139703 | -2.625977222 |
| tax_unhealthy_foods_lambda_meat_free_diet | 0 | NA | NA |
| tax_unhealthy_foods_lambda_govt_int_health | 0 | NA | NA |
| tax_high_sugar_foods_midpoint | 52.47314939 | 22.81345112 | 2.300096953 |
| tax_high_sugar_foods_range | 0 | NA | NA |
| tax_high_sugar_foods_over65 | 0 | NA | NA |
| tax_high_sugar_foods_under30 | 0 | NA | NA |
| tax_high_sugar_foods_female | 0 | NA | NA |
| tax_high_sugar_foods_nonwhite | 0 | NA | NA |
| tax_high_sugar_foods_heduc | 0 | NA | NA |
| tax_high_sugar_foods_urban | 0 | NA | NA |
| tax_high_sugar_foods_lambda_cc_belief | -35.9912056 | 14.89193477 | -2.416825358 |
| tax_high_sugar_foods_lambda_meat_free_diet | -21.61915966 | 10.17853602 | -2.123995005 |
| tax_high_sugar_foods_lambda_govt_int_health | 0 | NA | NA |
| tax_high_salt_foods_midpoint | 41.93497908 | 25.16052886 | 1.666697044 |
| tax_high_salt_foods_range | 0 | NA | NA |
| tax_high_salt_foods_over65 | 0 | NA | NA |
| tax_high_salt_foods_under30 | 0 | NA | NA |
| tax_high_salt_foods_female | 0 | NA | NA |
| tax_high_salt_foods_nonwhite | -43.52438717 | 19.85114281 | -2.192538112 |
| tax_high_salt_foods_heduc | 0 | NA | NA |
| tax_high_salt_foods_urban | 0 | NA | NA |
| tax_high_salt_foods_lambda_cc_belief | -37.65181473 | 16.05366563 | -2.345371804 |
| tax_high_salt_foods_lambda_meat_free_diet | -28.8486329 | 10.82331926 | -2.665414574 |
| tax_high_salt_foods_lambda_govt_int_health | 0 | NA | NA |
| subsidise_high_fibre_foods | 0 | NA | NA |
| subsidise_fresh_produce_midpoint | 9.276821189 | 8.451897626 | 1.097602172 |
| subsidise_fresh_produce_range | 39.58787131 | 20.96718453 | 1.888087132 |
| subsidise_fresh_produce_over65 | -24.03217684 | 15.27921619 | -1.572867125 |
| subsidise_fresh_produce_under30 | 0 | NA | NA |
| subsidise_fresh_produce_female | 0 | NA | NA |
| subsidise_fresh_produce_nonwhite | 0 | NA | NA |
| subsidise_fresh_produce_heduc | 0 | NA | NA |
| subsidise_fresh_produce_urban | 0 | NA | NA |
| subsidise_fresh_produce_lambda_cc_belief | 0 | NA | NA |
| subsidise_fresh_produce_lambda_meat_free_diet | 0 | NA | NA |
| subsidise_fresh_produce_lambda_govt_int_health | 0 | NA | NA |
| policy_affects_general_population | 0 | NA | NA |
| tax_affects_high_income_midpoint | -9.589193696 | 20.66870407 | -0.463947506 |
| tax_affects_high_income_range | -80.03040655 | 24.82522782 | -3.223753157 |
| tax_affects_high_income_over65 | 0 | NA | NA |
| tax_affects_high_income_under30 | 51.01023642 | 31.36812105 | 1.626180808 |
| tax_affects_high_income_female | -29.16894402 | 15.82688905 | -1.84299921 |
| tax_affects_high_income_nonwhite | 0 | NA | NA |
| tax_affects_high_income_heduc | -23.98205644 | 13.64284823 | -1.757848217 |
| tax_affects_high_income_urban | 0 | NA | NA |
| tax_affects_high_income_lambda_cc_belief | 27.71958691 | 13.45903575 | 2.059552217 |
| tax_affects_high_income_lambda_meat_free_diet | 0 | NA | NA |
| tax_affects_high_income_lambda_govt_int_health | -23.59955554 | 10.15395738 | -2.324173193 |
| subsidy_affects_low_income_midpoint | 38.08129671 | 17.16279948 | 2.218827805 |
| subsidy_affects_low_income_range | 0 | NA | NA |
| subsidy_affects_low_income_over65 | 0 | NA | NA |
| subsidy_affects_low_income_under30 | 0 | NA | NA |
| subsidy_affects_low_income_female | -21.13556705 | 16.41042136 | -1.287935671 |
| subsidy_affects_low_income_nonwhite | 28.88163458 | 21.0189492 | 1.374076044 |
| subsidy_affects_low_income_heduc | -33.8599472 | 17.77702575 | -1.904702602 |
| subsidy_affects_low_income_urban | 0 | NA | NA |
| subsidy_affects_low_income_lambda_cc_belief | 0 | NA | NA |
| subsidy_affects_low_income_lambda_meat_free_diet | 0 | NA | NA |
| subsidy_affects_low_income_lambda_govt_int_health | 0 | NA | NA |
| subsidy_affects_families_children_midpoint | 19.15425773 | 14.32313028 | 1.337295504 |
| subsidy_affects_families_children_range | 0 | NA | NA |
| subsidy_affects_families_children_over65 | 0 | NA | NA |
| subsidy_affects_families_children_under30 | 0 | NA | NA |
| subsidy_affects_families_children_female | 0 | NA | NA |
| subsidy_affects_families_children_nonwhite | 0 | NA | NA |
| subsidy_affects_families_children_heduc | -69.10347075 | 22.81211207 | -3.029244751 |
| subsidy_affects_families_children_urban | -29.20779494 | 18.65658024 | -1.565549235 |
| subsidy_affects_families_children_lambda_cc_belief | 0 | NA | NA |
| subsidy_affects_families_children_lambda_meat_free_diet | 18.9443475 | 10.49225909 | 1.805554681 |
| subsidy_affects_families_children_lambda_govt_int_health | 0 | NA | NA |
| subsidy_affects_overweight_obese_midpoint | -11.60014385 | 12.54473422 | -0.924702241 |
| subsidy_affects_overweight_obese_range | 46.32869732 | 24.5513185 | 1.887014635 |
| subsidy_affects_overweight_obese_over65 | 36.71406729 | 20.82274 | 1.763171768 |
| subsidy_affects_overweight_obese_under30 | 0 | NA | NA |
| subsidy_affects_overweight_obese_female | 0 | NA | NA |
| subsidy_affects_overweight_obese_nonwhite | 0 | NA | NA |
| subsidy_affects_overweight_obese_heduc | -47.35353344 | 19.72260091 | -2.40097813 |
| subsidy_affects_overweight_obese_urban | 0 | NA | NA |
| subsidy_affects_overweight_obese_lambda_cc_belief | 0 | NA | NA |
| subsidy_affects_overweight_obese_lambda_meat_free_diet | 0 | NA | NA |
| subsidy_affects_overweight_obese_lambda_govt_int_health | 0 | NA | NA |
| general_taxation | 0 | NA | NA |
| NHS_social_care_tax_midpoint | -43.4064795 | 20.90558012 | -2.076310691 |
| NHS_social_care_tax_range | 0 | NA | NA |
| NHS_social_care_tax_over65 | 0 | NA | NA |
| NHS_social_care_tax_under30 | 0 | NA | NA |
| NHS_social_care_tax_female | 40.25390207 | 18.11150235 | 2.222560078 |
| NHS_social_care_tax_nonwhite | 0 | NA | NA |
| NHS_social_care_tax_heduc | 0 | NA | NA |
| NHS_social_care_tax_urban | 0 | NA | NA |
| NHS_social_care_tax_lambda_cc_belief | 30.33362073 | 12.51356396 | 2.424059271 |
| NHS_social_care_tax_lambda_meat_free_diet | 0 | NA | NA |
| NHS_social_care_tax_lambda_govt_int_health | 0 | NA | NA |
| exercise_vouchers_tax_midpoint | -18.84404307 | 14.86942142 | -1.267301702 |
| exercise_vouchers_tax_range | 0 | NA | NA |
| exercise_vouchers_tax_over65 | -50.54269266 | 23.01547681 | -2.196030657 |
| exercise_vouchers_tax_under30 | -56.48057221 | 32.76267596 | -1.723930374 |
| exercise_vouchers_tax_female | 45.57428114 | 18.67102276 | 2.44090973 |
| exercise_vouchers_tax_nonwhite | 56.6922565 | 22.11552474 | 2.563459703 |
| exercise_vouchers_tax_heduc | -37.51051587 | 15.80764175 | -2.3729356 |
| exercise_vouchers_tax_urban | 0 | NA | NA |
| exercise_vouchers_tax_lambda_cc_belief | 0 | NA | NA |
| exercise_vouchers_tax_lambda_meat_free_diet | 0 | NA | NA |
| exercise_vouchers_tax_lambda_govt_int_health | 0 | NA | NA |
| NHS_social_care_subsidy_midpoint | -34.93490016 | 11.59526056 | -3.012860294 |
| NHS_social_care_subsidy_range | 0 | NA | NA |
| NHS_social_care_subsidy_over65 | 0 | NA | NA |
| NHS_social_care_subsidy_under30 | 0 | NA | NA |
| NHS_social_care_subsidy_female | 0 | NA | NA |
| NHS_social_care_subsidy_nonwhite | 28.07854096 | 16.23280886 | 1.729740133 |
| NHS_social_care_subsidy_heduc | 0 | NA | NA |
| NHS_social_care_subsidy_urban | 0 | NA | NA |
| NHS_social_care_subsidy_lambda_cc_belief | 0 | NA | NA |
| NHS_social_care_subsidy_lambda_meat_free_diet | 0 | NA | NA |
| NHS_social_care_subsidy_lambda_govt_int_health | 0 | NA | NA |
| exercise_vouchers_subsidy_midpoint | -1.582932422 | 11.89421188 | -0.133084263 |
| exercise_vouchers_subsidy_range | -37.06070964 | 21.57943589 | -1.71740864 |
| exercise_vouchers_subsidy_over65 | 0 | NA | NA |
| exercise_vouchers_subsidy_under30 | 0 | NA | NA |
| exercise_vouchers_subsidy_female | 0 | NA | NA |
| exercise_vouchers_subsidy_nonwhite | 0 | NA | NA |
| exercise_vouchers_subsidy_heduc | 31.37937932 | 16.80657346 | 1.867089648 |
| exercise_vouchers_subsidy_urban | 39.15031919 | 20.1908771 | 1.939010326 |
| exercise_vouchers_subsidy_lambda_cc_belief | 0 | NA | NA |
| exercise_vouchers_subsidy_lambda_meat_free_diet | -23.75390344 | 11.56583389 | -2.053799464 |
| exercise_vouchers_subsidy_lambda_govt_int_health | 0 | NA | NA |
| left_to_right_bias_dce2 | 0.075672401 | 0.0325665 | 2.323627058 |
| level_of_tax_midpoint | -4.843471285 | 0.240178317 | -20.16614718 |
| level_of_tax_range | 1.65024046 | 0.495862505 | 3.328020252 |
| lambda_income | -0.420402044 | 0.087152991 | -4.823724826 |
| level_of_tax_over65 | 0 | NA | NA |
| level_of_tax_under30 | -0.634797811 | 0.140815048 | -4.50802537 |
| level_of_tax_female | 0 | NA | NA |
| level_of_tax_nonwhite | 0 | NA | NA |
| level_of_tax_heduc | 0 | NA | NA |
| level_of_tax_urban | 0 | NA | NA |
| level_of_tax_lambda_cc_belief | 0 | NA | NA |
| level_of_tax_lambda_meat_free_diet | 1.017699884 | 0.115505925 | 8.810802415 |
| level_of_tax_lambda_govt_int_health | -0.156036756 | 0.07868733 | -1.982997214 |
| tax_water_use | 0 | NA | NA |
| tax_meat_dairy_midpoint | 22.45919776 | 4.951502333 | 4.535835035 |
| tax_meat_dairy_range | 0 | NA | NA |
| tax_meat_dairy_over65 | 0 | NA | NA |
| tax_meat_dairy_under30 | 0 | NA | NA |
| tax_meat_dairy_female | 0 | NA | NA |
| tax_meat_dairy_nonwhite | 0 | NA | NA |
| tax_meat_dairy_heduc | -6.495097346 | 3.129125889 | -2.075690649 |
| tax_meat_dairy_urban | 0 | NA | NA |
| tax_meat_dairy_lambda_cc_belief | 0 | NA | NA |
| tax_meat_dairy_lambda_meat_free_diet | -14.81783518 | 3.561361482 | -4.160722033 |
| tax_meat_dairy_lambda_govt_int_health | 8.617773085 | 2.131030482 | 4.043946419 |
| tax_greenhouse_gases_midpoint | 31.28328023 | 6.460183309 | 4.842475629 |
| tax_greenhouse_gases_range | 3.762441108 | 2.576794762 | 1.460124478 |
| tax_greenhouse_gases_over65 | 0 | NA | NA |
| tax_greenhouse_gases_under30 | 0 | NA | NA |
| tax_greenhouse_gases_female | 0 | NA | NA |
| tax_greenhouse_gases_nonwhite | 0 | NA | NA |
| tax_greenhouse_gases_heduc | -8.118213461 | 3.937336399 | -2.061854167 |
| tax_greenhouse_gases_urban | 0 | NA | NA |
| tax_greenhouse_gases_lambda_cc_belief | 5.075584016 | 3.025095335 | 1.677826136 |
| tax_greenhouse_gases_lambda_meat_free_diet | -28.31663143 | 3.777932397 | -7.495272138 |
| tax_greenhouse_gases_lambda_govt_int_health | 7.132863653 | 2.760859519 | 2.583566314 |
| tax_plastic_production_midpoint | 45.04968294 | 7.127586976 | 6.320467655 |
| tax_plastic_production_range | 6.63725516 | 2.897389904 | 2.290770445 |
| tax_plastic_production_over65 | 0 | NA | NA |
| tax_plastic_production_under30 | 0 | NA | NA |
| tax_plastic_production_female | 0 | NA | NA |
| tax_plastic_production_nonwhite | -18.30487593 | 5.755622267 | -3.180346986 |
| tax_plastic_production_heduc | 0 | NA | NA |
| tax_plastic_production_urban | 0 | NA | NA |
| tax_plastic_production_lambda_cc_belief | 5.277718769 | 2.905843742 | 1.816243142 |
| tax_plastic_production_lambda_meat_free_diet | -31.19157219 | 3.864422254 | -8.07147101 |
| tax_plastic_production_lambda_govt_int_health | 8.695552398 | 3.00767382 | 2.891122149 |
| low_impact_foods_midpoint | 9.252788438 | 4.43711325 | 2.085317168 |
| low_impact_foods_range | 0 | NA | NA |
| low_impact_foods_over65 | 0 | NA | NA |
| low_impact_foods_under30 | 0 | NA | NA |
| low_impact_foods_female | 13.05011451 | 3.707295107 | 3.520117533 |
| low_impact_foods_nonwhite | 0 | NA | NA |
| low_impact_foods_heduc | -4.78206066 | 3.524410692 | -1.356839789 |
| low_impact_foods_urban | 0 | NA | NA |
| low_impact_foods_lambda_cc_belief | 0 | NA | NA |
| low_impact_foods_lambda_meat_free_diet | -15.14616996 | 3.447233087 | -4.393717971 |
| low_impact_foods_lambda_govt_int_health | 6.506606667 | 2.175762911 | 2.990494338 |
| UK_environment_midpoint | 3.435739869 | 4.372534745 | 0.785754732 |
| UK_environment_range | 0 | NA | NA |
| UK_environment_over65 | 0 | NA | NA |
| UK_environment_under30 | 0 | NA | NA |
| UK_environment_female | 8.446193505 | 2.981383263 | 2.832978104 |
| UK_environment_nonwhite | 0 | NA | NA |
| UK_environment_heduc | -5.123722992 | 2.937812366 | -1.744060666 |
| UK_environment_urban | 0 | NA | NA |
| UK_environment_lambda_cc_belief | 4.56898803 | 2.309866915 | 1.978030856 |
| UK_environment_lambda_meat_free_diet | -12.48241008 | 2.46539343 | -5.063049948 |
| UK_environment_lambda_govt_int_health | 0 | NA | NA |
| donate_midpoint | -75.22825766 | 6.361586016 | -11.82539346 |
| donate_range | -5.885082339 | 2.674159729 | -2.200722072 |
| donate_over65 | -6.802972341 | 4.871777344 | -1.39640461 |
| donate_under30 | 11.74192662 | 9.137882075 | 1.28497244 |
| donate_female | 0 | NA | NA |
| donate_nonwhite | 36.52038437 | 5.448912204 | 6.702325712 |
| donate_heduc | 0 | NA | NA |
| donate_urban | -11.86003176 | 8.003711033 | -1.481816586 |
| donate_lambda_cc_belief | 0 | NA | NA |
| donate_lambda_meat_free_diet | 30.22956845 | 2.807640666 | 10.76689365 |
| donate_lambda_govt_int_health | -10.80191176 | 3.181098177 | -3.395654947 |
| gamma_cc_belief_over65 | 1.012524452 | 0.096111709 | 10.53487093 |
| gamma_cc_belief_under30 | 0.331731156 | 0.064388029 | 5.152062594 |
| gamma_cc_belief_female | 1.184693569 | 0.05911991 | 20.03882565 |
| gamma_cc_belief_nonwhite | 0.211996394 | 0.068553781 | 3.09240995 |
| gamma_cc_belief_heduc | 0.720905421 | 0.072051223 | 10.0054571 |
| gamma_cc_belief_urban | 0.353958871 | 0.067668207 | 5.230800168 |
| delta_believe_cc | -5 | NA | NA |
| zeta_believe_cc | 11.60691322 | 0.655571696 | 17.70502492 |
| zeta_worry_cc | 4.823889428 | 0.312867767 | 15.41830107 |
| tau_worry_cc_1 | 2.521777863 | 0.258052822 | 9.772332068 |
| tau_worry_cc_2 | 4.732589457 | 0.358163363 | 13.21349403 |
| tau_worry_cc_3 | 9.047149057 | 0.55778067 | 16.21990425 |
| tau_worry_cc_4 | 11.68409627 | 0.67941633 | 17.19725558 |
| zeta_responsibility_cc | 3.946653578 | 0.080090842 | 49.27721425 |
| sigma_responsibility_cc | 1.655907922 | 0.05910338 | 28.01714404 |
| zeta_pay_env_friendly_prods | 1.80089166 | 0.112215315 | 16.04853726 |
| tau_pay_env_friendly_prods_1 | 0.032774952 | 0.163001639 | 0.201071302 |
| tau_pay_env_friendly_prods_2 | 1.603642706 | 0.165952003 | 9.663292239 |
| tau_pay_env_friendly_prods_3 | 3.159196373 | 0.185765712 | 17.00634814 |
| tau_pay_env_friendly_prods_4 | 6.14442978 | 0.237262071 | 25.89722729 |
| zeta_govt_int_env | 1.555899759 | 0.113954343 | 13.65371178 |
| tau_govt_int_env_1 | -2.223269583 | 0.207469349 | -10.71613514 |
| tau_govt_int_env_2 | -1.207572149 | 0.18109862 | -6.668036159 |
| tau_govt_int_env_3 | 0.503479135 | 0.170107623 | 2.959768212 |
| tau_govt_int_env_4 | 3.384422451 | 0.203844117 | 16.602993 |
| gamma_meat_free_diet_over65 | 0.281007581 | 0.098083421 | 2.864985529 |
| gamma_meat_free_diet_under30 | -0.302247001 | 0.08546885 | -3.536341025 |
| gamma_meat_free_diet_female | 0 | NA | NA |
| gamma_meat_free_diet_nonwhite | -0.527385024 | 0.091904475 | -5.738404195 |
| gamma_meat_free_diet_heduc | 0 | NA | NA |
| gamma_meat_free_diet_urban | 0 | NA | NA |
| delta_veg_vegan | -8.012320903 | 1.167098442 | -6.865162883 |
| zeta_veg_vegan | -5.346179255 | 1.085449096 | -4.925315499 |
| delta_eats_Dairy | 3.147824279 | 0.200769607 | 15.67878884 |
| zeta_eats_Dairy | 1.928408518 | 0.191100911 | 10.09104829 |
| delta_eats_RedMeat | 1.891349531 | 0.157243154 | 12.0281836 |
| zeta_eats_RedMeat | 2.055642934 | 0.194916664 | 10.54626575 |
| delta_eats_WhiteMeat | 2.975333577 | 0.28603569 | 10.40196619 |
| zeta_eats_WhiteMeat | 3.050476645 | 0.303555552 | 10.04915452 |
| delta_eats_Fish | 1.675545074 | 0.121101854 | 13.83583337 |
| zeta_eats_Fish | 1.527304464 | 0.155707337 | 9.808814997 |
| delta_eats_NoneOfThese | -8.953182134 | 1.237424162 | -7.235338059 |
| zeta_eats_NoneOfThese | -4.13451605 | 0.739491193 | -5.591028116 |
| gamma_gov_health_over65 | 0 | NA | NA |
| gamma_gov_health_under30 | -0.145658839 | 0.062617351 | -2.326173768 |
| gamma_gov_health_female | 0 | NA | NA |
| gamma_gov_health_nonwhite | -0.144102206 | 0.071985755 | -2.001815575 |
| gamma_gov_health_heduc | 0.279711999 | 0.069420252 | 4.029256449 |
| gamma_gov_health_urban | 0 | NA | NA |
| zeta_pay_env_friendly_prods2 | -0.212207866 | 0.064715049 | -3.279111579 |
| zeta_govt_int_health | 1.361879889 | 0.105963214 | 12.85238373 |
| tau_govt_int_health_1 | -3.639794242 | 0.149108196 | -24.41042368 |
| tau_govt_int_health_2 | -2.149767266 | 0.111642098 | -19.25588381 |
| tau_govt_int_health_3 | -0.463078381 | 0.085901368 | -5.390814984 |
| tau_govt_int_health_4 | 2.21573232 | 0.116775619 | 18.97427173 |
| zeta_govt_int_env2 | 0.724708825 | 0.099385894 | 7.291868043 |
| zeta_govt_tax_sugary_drinks | 2.945072364 | 0.198946743 | 14.8033203 |
| tau_govt_tax_sugary_drinks_1 | -4.6990442 | 0.260316964 | -18.05124079 |
| tau_govt_tax_sugary_drinks_2 | -2.60943404 | 0.188489202 | -13.84394444 |
| tau_govt_tax_sugary_drinks_3 | -0.407499505 | 0.150377191 | -2.709849162 |
| tau_govt_tax_sugary_drinks_4 | 3.392370813 | 0.212786362 | 15.94261386 |
| zeta_govt_tax_sugary_drinks_health_spending | 3.795150371 | 0.279591968 | 13.57388912 |
| tau_govt_tax_sugary_drinks_health_spending_1 | -6.174638807 | 0.383816152 | -16.0874908 |
| tau_govt_tax_sugary_drinks_health_spending_2 | -3.958012318 | 0.279651223 | -14.15338821 |
| tau_govt_tax_sugary_drinks_health_spending_3 | -1.356591164 | 0.201619225 | -6.728481203 |
| tau_govt_tax_sugary_drinks_health_spending_4 | 3.194570081 | 0.264203633 | 12.09131775 |

Estimate – estimated coefficient; Rob.std.err – robust standard error; Rob.t.ratio(0) – robust t-ratio versus 0.

## Appendix C: Weighted versus non-weighted model

Health DCE

Sustainability DCE

## Appendix D: Data quality supplemental statistics

| *Invited participants* |  |  |
| --- | --- | --- |
| Total |  | 8420 |
|  |  |  |
| *Started the survey* |  |  |
| Total |  | 5140 |
|  |  |  |
| *Participants removed* |  |  |
| Failed eligibility |  | 83 |
| Timed out |  | 955 |
| Failed speeding checks |  | 15 |
|  |  |  |
| *Exceed quotas* |  |  |
| Over quota |  | 2067 |
|  |  |  |
| Final sample |  | 2020 |
|  |  |  |

Table A1: sampling statistics

## Appendix E: Descriptive statistics for sample and indicator variables used in the latent variables

| **Variable** | **N** |  | **Sample Mean** |  | **UK average** |
| --- | --- | --- | --- | --- | --- |
|  |  |  |  |  |  |
|  |  |  |  |  |  |
| Age: under 35 | 913 |  | 0.45 |  | 0.28 |
| Age: 35 to 64 | 824 |  | 0.41 |  | 0.49 |
| Age: Over 64 | 283 |  | 0.10 |  | 0.23 |
|  |  |  |  |  |  |
| Female | 1027 |  | 0.51 |  | 0.51 |
|  |  |  |  |  |  |
| Ethnic group: Non-white | 452 |  | 0.22 |  | 0.16 |
| Ethnic group: White | 1568 |  | 0.78 |  | 0.84 |
|  |  |  |  |  |  |
| Region: East England | 170 |  | 8.42 |  | 9.50 |
| Region: East Midlands | 154 |  | 7.62 |  | 7.30 |
| Region: London | 295 |  | 14.60 |  | 13.10 |
| Region: North East | 85 |  | 4.21 |  | 3.90 |
| Region: North West | 200 |  | 9.90 |  | 11.10 |
| Region: Northern Ireland | 39 |  | 1.93 |  | 2.80 |
| Region: Scotland | 151 |  | 7.48 |  | 8.20 |
| Region: South East | 298 |  | 14.80 |  | 13.90 |
| Region: South West | 173 |  | 8.56 |  | 8.50 |
| Region: Wales | 96 |  | 4.75 |  | 4.60 |
| Region: West Midlands | 190 |  | 9.41 |  | 8.90 |
| Region: Yorkshire and the Humber | 169 |  | 8.37 |  | 8.20 |
|  |  |  |  |  |  |
| Higher Education | 897 |  | 0.44 |  | 0.34 |
|  |  |  |  |  |  |
| Urban | 454 |  | 0.23 |  | 0.79 |
| Suburban | 957 |  | 0.47 |  | 0.79 |
| Rural | 609 |  | 0.30 |  | 0.21 |
|  |  |  |  |  |  |
| Relative low income | 497 |  | 0.25 |  | 0.14 |
|  |  |  |  |  |  |

Table A2: sample demographics. Sources of information for the UK average were gathered from the UK Office for National Statistics.

## Appendix F: Rationale for the use of latent variables

Whereas interacting individual characteristics is straightforward and considered standard practice, interacting the responses to attitudinal/lifestyle questions raises a number of issues (Ben-Akiva et al., 2022). It is not clear which of the variables should enter the utility function. Using all of them together may induce collinearity; yet using a subset means information in the data may be lost. This approach proliferates parameters, and models quickly become unwieldy. It is also not clear how these questions are able to capture the underlying concept of interest; that is, they are indirect, imperfect measures, e.g., of responsibility for climate change. They may also be subject to varying interpretation (one person’s score of 6 on a scale of 1 to 10 may be another person’s 7 or 8), which may result in measurement error.

Using latent variables within an integrated choice and latent variable framework (Walker, 2001) can overcome the issue that the variables are not direct measures of the concept of interest, by treating the attitudinal/lifestyle variables as indicators of an underlying latent variable. Their function is to explain utility (and ultimately choices) and to explain all of the attitudinal/lifestyle variables at the same time (by doing so, they are able to use all of the information in the data). They thus reduce the dimensionality of the choice model because only one variable is inserted into the utility function for each latent variable (as opposed to multiple questions individually). In addition, they avoid issues of collinearity that would likely arise if the attitudinal/lifestyle variables would enter together in the utility function. In addition, it is possible to explain the latent variables with individual characteristics, which is useful behavioural information.

## Appendix G: Description of scoping review methods

The aim of this scoping review was to identify, describe and characterise all health or environment subsidy and tax (HEST) food policies that have either been implemented (worldwide), or proposed by policymakers, NGOs, think-tanks or academics.

**Definitions**

For this scoping review protocol we defined HEST food policies as follows: *Proposed or implemented fiscal policies that directly alter the retail cost of food and non-alcoholic drinks and which are designed to improve health or reduce the environmental burden of the food system.*

HEST food policies may affect all of the population or be targeted to subgroups of the population. They may target specific food categories (e.g. confectionery) or be applied based on the properties of foods (e.g. salt content; greenhouse gas emissions) or both.

The definition includes *“directly alter the retail price”.* Here, we refer to policies that directly target food and non-alcoholic drink products or target companies based on volume sales of food and non-alcoholic drink products (e.g. the UK Soft Drink Industry Levy). We did not include taxes or subsidies targeting other activities of food and drink companies (e.g. advertising) that may result indirectly in changes of retail price of foods and non-alcoholic drinks.

**Inclusion and exclusion criteria**

Table 1 Inclusion and exclusion criteria

| **Characteristic** | **Inclusion criteria** | **Exclusion criteria** |
| --- | --- | --- |
| Year of publication | Published 2010 onwards. | Published before 2010 |
| Type of policy | Proposed or implemented fiscal policies that directly alter the retail cost of food and non-alcoholic drinks and which are designed to improve health or reduce the environmental burden of the food system e.g., vouchers for healthy food, taxes on sugar or sugary products (like soft drinks), negative VAT rates applied to targeted healthy food categories. | Policies not directly altering the retail cost of food and/or not designed to improve health or reduce the environmental burden of the food system e.g., free school meals, tariffs applied on imports unless specifically health-related, agricultural subsidies not aimed towards specific commodities. |
| Type of study | Database of implemented food fiscal policies: all records.  Academic literature: reviews from academic peer-reviewed literature that include a description of the method for searching/screening the literature; HEST policies should be the *main focus* of the review.  Policy document database: any publication; HEST policies should be the main focus of the publication. | Database of implemented food policies: none.  Academic literature: reviews that do not include a description of the method for searching/screening the literature; any other study design (e.g., commentary, modelling study); HEST policies are not the *main focus* of the review.  Policy document database: any study type other than publication (e.g., working paper, poster); HEST policies are not the main focus of the publication. |
| Language | Published in any language |  |
| Country | Any |  |

**Search strategy**

We used key terms to search sequentially: (i) a database of implemented food policies, (ii) academic literature databases, and grey literature using (iii) a policy document database.

**Database of implemented food policies**

We hand-searched the World Cancer Research Forum NOURISHING database (available at <https://policydatabase.wcrf.org/>) in May 2022 and retrieved all entries listed under the tab “Use economic tools to address food affordability and purchase incentives”.

**Academic literature**

We searched five databases from database inception to 27^th^ September 2022: Medline (OvidSP)[1946-present], CAB Abstracts (OvidSP)[2000 to 2022 Week 38], Scopus (Elsevier), CEE Database of Evidence Reviews (CEEDER)[https://environmentalevidence.org/ceeder/] and Epistemonikos [https://www.epistemonikos.org/en/]. We combined author keywords and subject headings for food, beverages or environmental phenomena and taxes, incentives or subsidies. We applied a customised search filter for systematic reviews based on Navarro-Ruan and Haynes, combined with evidence syntheses keywords from the Collaboration for Environmental Evidence. We limited the search from 2010 onwards, and did not apply language limits. Full search strategies available in **Appendix H.**

**Policy document database search**

Overton [<https://www.overton.io/>] is the world’s largest searchable index of policy documents, guidelines, think tank publications and working papers. We searched Overton from inception to the 15^th^ November 2022, using key terms, and limited the search to publication type “publication” and to results published since 1^st^ January 2010.

**Screening**

**Database of implemented food policies**

We undertook no formal screening for this data source as we assumed all entries under “Use economic tools to address food affordability and purchase incentives” in the NOURISHING database were relevant.

**Academic literature**

Screening was conducted via the inbuilt function in Endnote, reference management software, and then in Covidence, software for managing systematic review (available at: <https://www.covidence.org/>), we removed duplicate results of the database searches. Authors (JB, LB, MB, HF, AK) first tested the draft exclusion criteria by screening a randomly selected sample of publications (n=65, 5%), met to discuss the outcome, and refined the exclusion criteria (resultant criteria presented in **Table 1**). Two authors then simultaneously, independently screened the titles and abstracts for each record (equally divided between JB, LB, MB, HF, AK). We excluded records for which both screeners agreed did not meet the eligibility criteria. All records that had a conflicting decision between screeners advanced to full text review to minimise risk of excluding potentially relevant texts. We retrieved full texts of the remaining records using the embedded function in Covidence or by hand. Finally, full texts were then screened by HF.

**Policy document database**

The same researchers tested draft exclusion criteria by screening a random sample of publications (n=56, 5%), and then met to discuss the outcome. The key change was not to apply criteria relating to publication type, because policy documents rarely include literature review supported by a method. The same authors (JB, LB, MB, HF, AK) simultaneously, independently screened the titles and abstracts of the titles and abstracts for each record, and then the full texts of those passing title and abstract screening.

**Data extraction**

Our unit of interest was distinct HEST policies, stratified by country (i.e. identical HEST policies identified in multiple countries would be considered as multiple HEST policies). We extracted data additively to reduce the risk of extracting duplicative policies: firstly from the database of implemented food policies, then from the academic literature, and finally from the policy document dataset. We recorded the data in an excel spreadsheet whereby each HEST policy was documented in a row. We recorded details that addressed our research objectives in each column, specifically: publication information (e.g., author, publication year, publication type), information about the HEST policy (description of subsidy or tax, year of implementation or appeal, setting, targeted population, targeted food categories).

**Synthesising policies**

Policies were synthesised through aggregating policies with identical attributes. This was achieved by creating dummy variables for the attributes in Table 2. The resulting binary dataset was then used to aggregate policies based on unique combinations of these attributes. An R package, UpsetR^[1]^, was used to visualise the intersections of different HEST policy attributes.

**Table 2 Encoding of policy attributes**

| **Policy attributes** | **Related binary variables** |
| --- | --- |
| Policy type | Tax, subsidy, tax and subsidy |
| Implementation status | Implemented, proposed |
| Policy scope | Population (untargeted), high-risk (targeted) |
| Target population | Individuals with low income, recipients of benefits, general population, patients, women/pregnant women/children/families |
| Targeted food group(s) | Bread, confectionary, dairy, fish, fresh produce, fruit and vegetables, fruit juice,  healthy/unhealthy as defined by a nutrient profile model, ice cream, meat, soft drinks |
| Targeted nutrient(s)/component(s) | Calcium, calories, fat, fibre, greenhouse gases, high fructose corn syrup, msg, non-nutritive sweeteners, salt, saturated fat, sugar, trans fat |
| Tax/Subsidy levels | Dummy variables created for percentage bands and amounts |
| Countries | Dummy variables created for 112 countries/states |

**Results**

**Summary of included literature**

**Figure X** summarises the search and screening results. In total, we retrieved 105 records from the database of implemented food policies, 1,773 records from academic literature, and 1,127 records from the policy document database. We identified and removed 480 duplicate records in the academic literature. We retrieved and screened the full texts of 389 records from academic literature and 82 records from the policy document database. In total, details for 753 HEST policies were recorded.

**Summary of HEST policy characteristics**

Of the total extracted policies (n =753), 521 discrete policies were identified by grouping policies with identical attributes, of which 414 were taxes, 127 were subsidies, and 27 were policies that combined tax and subsidies. There were 207 policies that have been implemented, of which 197 were taxes and 10 were subsidies. In total, 304 policies did not target a specific population group (i.e. policies aimed at the general population) and 28 polices targeted a particular group. The most common target population group was women, children and families.

Policies were identified in 93 countries: 134 policies originated from the USA, the next most common country was the UK (31 policies), followed by Australia and France (18 policies each) and Mexico (n=15). A single HEST policy was identified in 27 countries.

The most common target food group, that is the food group that is altered by the HEST policy, was soft drinks (n=304 policies), followed by fruits and vegetables (n=69), dairy products (n=18) and fruit juice (n=14). Meat, unhealthy foods (as defined by a nutrient profile model), confectionery appeared in fewer than ten policies.

The most common target nutrient was sugar (100 policies), followed by fat (n=31), saturated fat (n=21), energy/calories (n=20), and salt/sodium (18 policies).

Of policies that were levied as a percentage (n=267 policies), the most common percentage applied was 21-50% (97 policies), 10% and less (84 policies), 11to20% (n=72).

Figure 2 shows the most common combination of attributes.

 Figure 1 PRISMA chart

**Strengths and limitations of approach**

A strength of this scoping review is that it considered peer-reviewed academic literature and documents produced by governments, NGOs, and think tanks to give a comprehensive overview of HEST policies. The databases searched included a policy document database that contains documents from 188 countries and over 1000 sources and a database of policies that have been implemented in the real world. The synthesis of proposed and implemented HEST policies means that the resulting policy attributes are feasible and realistic HEST policy features.

Our scoping review was not intended to be an exhaustive search for HEST policies. As we only included published reviews in academic literature, we may have overlooked HEST policies reported in other types of publications e.g., primary studies. Triangulating literature sources helped to mitigate the risks of overlooking major policies. Our sources (e.g., NOURISHING) were inherently more focused on health than environmental policies, which could mean we have under-represented environment-related policies in our resultant dataset. There may be other HEST policies relevant to-, but not specifically for-, food that were not captured in this review e.g. welfare or agricultural policies.

**Figure 2**: UpsetR diagram showing the most common intersections/combination of attributes. The horizontal graph displays the “frequency of attribute combinations” and the vertical graph displays the frequency of each attribute without any cross tabulations. This figure shows, for example, that 414 policies were taxes, of which 39 were implemented tax policies applied to soft drinks based on their sugar content, and targeted the the general population.

## Appendix H: Full literature search strategy

Medline (OvidSP) [1946-present]

| **Search number** | **Search terms** | **Hits** |
| --- | --- | --- |
| 1 | taxes/ or tax exemption/ | 8314 |
| 2 | (tax or taxes or taxation or levy or levies).ti. | 8206 |
| 3 | ((fiscal or economic or financial) adj2 (policy or plan* or lever*)).ti,ab,kf. | 3616 |
| 4 | financing, government/ or food assistance/ or public expenditures/ | <https://ezproxy-prd.bodleian.ox.ac.uk:2483/ovid-a/ovidweb.cgi?&S=DGLOFPGFPGEBIFGIJPPJBHBFJPFIAA00&R=19&Search+Annotations+Options=SA>23018 |
| 5 | (subsid* or voucher? or incentive? or discount*).ti. | 11889 |
| 6 | 1 or 2 or 3 or 4 or 5 | 50337 |
| 7 | environmental policy/ or exp nutrition policy/ | 15632 |
| 8 | environmental pollutants/ or air pollutants/ or greenhouse gases/ or exp water pollutants/ | 234143 |
| 9 | environmental pollution/ or air pollution/ or exp "environmental restoration and remediation"/ or exp water pollution/ | <https://ezproxy-prd.bodleian.ox.ac.uk:2483/ovid-a/ovidweb.cgi?&S=DGLOFPGFPGEBIFGIJPPJBHBFJPFIAA00&R=29&Search+Annotations+Options=SA><https://ezproxy-prd.bodleian.ox.ac.uk:2483/ovid-a/ovidweb.cgi?&S=DGLOFPGFPGEBIFGIJPPJBHBFJPFIAA00&R=31&Search+Annotations+Options=SA>138512 |
| 10 | exp climate change/ or greenhouse effect/ or carbon footprint/ | 33342 |
| 11 | biodiversity/ | 39531 |
| 12 | (environment* or (climat* adj change) or global warming or greenhouse gas or green or ghg? or carbon footprint? or pollut* or biodivers*).ti. | 301395 |
| 13 | ((environment* or (climat* adj change) or global warming or greenhouse gas or green or ghg? or carbon footprint? or pollut* or biodivers*) adj5 (policy or policies or strateg* or framework?)).ti,ab,kf. | 27158 |
| 14 | beverages/ or artificially sweetened beverages/ or exp carbonated beverages/ or energy drinks/ or sugar-sweetened beverages/ or exp food/ | 1435608 |
| 15 | (food or fat? or sugar? or salt or meat or fruit or vegetable?).ti. | 342379 |
| 16 | (beverage? or soda? or ((carbonated or sugar-sweetened or energy or soft) adj drink?)).ti. | 11254 |
| 17 | 7 or 8 or 9 or 10 or 11 or 12 or 13 or 14 or 15 or 16 | 2215328 |
| 18 | 6 and 17 | 4022 |
| 19 | ((carbon or green* or climat* or environment* or biodivers*) adj3 (tax or taxes or taxation or levy or levies)).ti,ab,kf. | 421 |
| 20 | ((carbon or green* or climat* or environment* or biodivers*) adj3 (subsid* or voucher? or incentive? or discount*)).ti,ab,kf. | 508 |
| 21 | ((food or fat? or sugar? or salt or meat or fruit or vegetable?) adj3 (tax or taxes or taxation or levy or levies)).ti,ab,kf. | 704 |
| 22 | ((food or fat? or sugar? or salt or meat or fruit or vegetable?) adj3 (subsid* or voucher? or incentive? or discount*)).ti,ab,kf. | 933 |
| 23 | ((beverage? or soda? or drink?) adj3 (tax or taxes or taxation or levy or levies)).ti,ab,kf. | 557 |
| 24 | ((beverage? or soda? or drink?) adj3 (subsid* or voucher? or incentive? or discount*)).ti,ab,kf. | 41 |
| 25 | 18 or 19 or 20 or 21 or 22 or 23 or 24 | 5577 |
| 26 | (systematic review or meta-analysis or review).pt. | 3200070 |
| 27 | (search* or meta analysis).ti,ab. or meta analysis/ | 700130 |
| 28 | (scoping review or (evidence adj2 (review or synthesis or map*))).ti,ab,kf. | 49309 |
| 29 | 26 or 27 or 28 | 3518225 |
| 30 | 25 and 29 | 602 |
| 31 | **limit 30 to yr="2010 -Current"** | **491** |

CAB Abstracts (OvidSP) [2000 to 2022 Week 38]

| **Search number** | **Search terms** | **Hits** |
| --- | --- | --- |
| 1 | taxes/ or value added tax/ or tax incentives/ or levies/ | 4588 |
| 2 | (tax or taxes or taxation or levy or levies).ti. | 2381 |
| 3 | ((fiscal or economic or financial) adj2 (policy or plan* or lever*)).ti,ab. | 4621 |
| 4 | fiscal policy/ or public expenditure/ or public finance/ | 2669 |
| 5 | incentives/ or subsidies/ | 13066 |
| 6 | (subsid* or voucher? or incentive? or discount*).ti. | 5973 |
| 7 | 1 or 2 or 3 or 4 or 5 or 6 | 26767 |
| 8 | environmental policy/ or food policy/ or nutrition policy/ or price policy/ | 20032 |
| 9 | pollutants/ or exp air pollutants/ or pollution/ or exp air pollution/ or exp water pollution/ | <https://ezproxy-prd.bodleian.ox.ac.uk:2483/ovid-a/ovidweb.cgi?&S=DGLOFPGFPGEBIFGIJPPJBHBFJPFIAA00&R=29&Search+Annotations+Options=SA>304111 |
| 10 | exp environmental impact/ | <https://ezproxy-prd.bodleian.ox.ac.uk:2483/ovid-a/ovidweb.cgi?&S=DGLOFPGFPGEBIFGIJPPJBHBFJPFIAA00&R=31&Search+Annotations+Options=SA>69983 |
| 11 | exp climate change/ or greenhouse effect/ | 146002 |
| 12 | biodiversity/ | 84328 |
| 13 | (environment* or (climat* adj change) or global warming or greenhouse gas or green or ghg? or carbon footprint? or pollut* or biodivers*).ti. | 287325 |
| 14 | ((environment* or (climat* adj change) or global warming or greenhouse gas or green or ghg? or carbon footprint? or pollut* or biodivers*) adj5 (policy or policies or strateg* or framework?)).ti,ab. | 38272 |
| 15 | beverages/ or artificially sweetened beverages/ or exp carbonated beverages/ or energy drinks/ or sugar-sweetened beverages/ or exp food/ | 403900 |
| 16 | (food or fat? or sugar? or salt or meat or fruit or vegetable?).ti. | 381979 |
| 17 | (beverage? or soda? or ((carbonated or sugar-sweetened or energy or soft) adj drink?)).ti. | 12069 |
| 18 | 8 or 9 or 10 or 11 or 12 or 13 or 14 or 15 or 16 or 17 | 1357967 |
| 19 | 7 and 18 | 7920 |
| 20 | ((carbon or green* or climat* or environment* or biodivers*) adj3 (tax or taxes or taxation or levy or levies)).ti,ab. | 908 |
| 21 | ((carbon or green* or climat* or environment* or biodivers*) adj3 (subsid* or voucher? or incentive? or discount*)).ti,ab. | 1397 |
| 22 | ((food or fat? or sugar? or salt or meat or fruit or vegetable?) adj3 (tax or taxes or taxation or levy or levies)).ti,ab. | 611 |
| 23 | ((food or fat? or sugar? or salt or meat or fruit or vegetable?) adj3 (subsid* or voucher? or incentive? or discount*)).ti,ab. | 1209 |
| 24 | ((beverage? or soda? or drink?) adj3 (tax or taxes or taxation or levy or levies)).ti,ab. | 389 |
| 25 | ((beverage? or soda? or drink?) adj3 (subsid* or voucher? or incentive? or discount*)).ti,ab. | 28 |
| 26 | 19 or 20 or 21 or 22 or 23 or 24 or 25 | 10230 |
| 27 | reviews/ or scoping reviews/ or systematic reviews/ | 253991 |
| 28 | (search* or meta analysis).ti,ab. | 116181 |
| 29 | (scoping review or (evidence adj2 (review? or synthes?s or map*))).ti,ab. | 6437 |
| 30 | 27 or 28 | <https://ezproxy-prd.bodleian.ox.ac.uk:2483/ovid-a/ovidweb.cgi?&S=DGLOFPGFPGEBIFGIJPPJBHBFJPFIAA00&R=6&Search+Annotations+Options=SA>338112 |
| 31 | 26 and 30 | 487 |
| 32 | **limit 31 to yr="2010 -Current"** | **<https://ezproxy-prd.bodleian.ox.ac.uk:2483/ovid-a/ovidweb.cgi?&S=DGLOFPGFPGEBIFGIJPPJBHBFJPFIAA00&R=10&Search+Annotations+Options=SA>385** |

Scopus (Elsevier)

| **Search terms** | **Hits** |
| --- | --- |
| ( ( TITLE-ABS-KEY ( ( carbon  OR  green*  OR  climat*  OR  environment*  OR  biodivers* )  W/3  ( tax  OR  taxes  OR  taxation  OR  levy  OR  levies ) )  OR  TITLE-ABS-KEY ( ( carbon  OR  green*  OR  climat*  OR  environment*  OR  biodivers* )  W/3  ( subsid*  OR  voucher*  OR  incentive*  OR  discount* ) )  OR  TITLE-ABS-KEY ( ( food  OR  fat*  OR  sugar*  OR  salt  OR  meat  OR  fruit  OR  vegetable* )  W/3  ( tax  OR  taxes  OR  taxation  OR  levy  OR  levies ) )  OR  TITLE-ABS-KEY ( ( food  OR  fat*  OR  sugar*  OR  salt  OR  meat  OR  fruit  OR  vegetable* )  W/3  ( subsid*  OR  voucher*  OR  incentive*  OR  discount* ) )  OR  TITLE-ABS-KEY ( ( beverage*  OR  soda*  OR  drink* )  W/3  ( tax  OR  taxes  OR  taxation  OR  levy  OR  levies ) )  OR  TITLE-ABS-KEY ( ( beverage*  OR  soda*  OR  drink* )  W/3  ( subsid*  OR  voucher*  OR  incentive*  OR  discount* ) ) )  OR  ( ( ( TITLE ( food  OR  fat*  OR  sugar*  OR  salt  OR  meat  OR  fruit  OR  vegetable* )  OR  TITLE ( beverage*  OR  soda*  OR  ( ( carbonated  OR  sugar-sweetened  OR  energy  OR  soft )  W/1  drink* ) ) )  OR  ( INDEXTERMS ( beverages )  OR  INDEXTERMS ( "artificially sweetened beverages" )  OR  INDEXTERMS ( "carbonated beverages" )  OR  INDEXTERMS ( "energy drinks" )  OR  INDEXTERMS ( "sugar-sweetened beverages" )  OR  INDEXTERMS ( food ) )  OR  ( TITLE ( environment*  OR  ( climat*  W/1  change )  OR  "global warming"  OR  "greenhouse gas"  OR  green  OR  ghg*  OR  "carbon footprint*"  OR  pollut*  OR  biodivers* )  OR  TITLE-ABS-KEY ( ( environment*  OR  ( climat*  W/1  change )  OR  "global warming"  OR  "greenhouse gas"  OR  green  OR  ghg*  OR  "carbon footprint*"  OR  pollut*  OR  biodivers* )  W/5  ( policy  OR  policies  OR  strateg*  OR  framework* ) ) )  OR  ( INDEXTERMS ( "environmental policy" )  OR  INDEXTERMS ( "nutrition policy" )  OR  INDEXTERMS ( "environmental pollutants" )  OR  INDEXTERMS ( "air pollutants" )  OR  INDEXTERMS ( "greenhouse gases" )  OR  INDEXTERMS ( "water pollutants" )  OR  INDEXTERMS ( "environmental pollution" )  OR  INDEXTERMS ( "air pollution" )  OR  INDEXTERMS ( "environmental restoration and remediation" )  OR  INDEXTERMS ( "water pollution" )  OR  INDEXTERMS ( "climate change" )  OR  INDEXTERMS ( "greenhouse effect" )  OR  INDEXTERMS ( "carbon footprint" ) ) )  AND  ( ( TITLE ( subsid*  OR  voucher*  OR  incentive*  OR  discount* ) )  OR  ( INDEXTERMS ( "financing, government" )  OR  INDEXTERMS ( "food assistance" )  OR  INDEXTERMS ( "public expenditures" ) )  OR  ( TITLE-ABS-KEY ( ( fiscal  OR  economic  OR  financial )  W/2  ( policy  OR  plan*  OR  lever* ) ) )  OR  ( TITLE ( tax  OR  taxes  OR  taxation  OR  levy  OR  levies ) )  OR  ( INDEXTERMS ( taxes )  OR  INDEXTERMS ( "tax exemption" ) ) ) ) )  AND  ( TITLE-ABS ( search* )  OR  INDEXTERMS ( "meta analysis" )  OR  TITLE ( "scoping review"  OR  "systematic review"  OR  "meta analysis"  OR  ( evidence  PRE/2  ( review  OR  synthesis  OR  map* ) ) ) )  AND  ( LIMIT-TO ( PUBYEAR ,  2022 )  OR  LIMIT-TO ( PUBYEAR ,  2021 )  OR  LIMIT-TO ( PUBYEAR ,  2020 )  OR  LIMIT-TO ( PUBYEAR ,  2019 )  OR  LIMIT-TO ( PUBYEAR ,  2018 )  OR  LIMIT-TO ( PUBYEAR ,  2017 )  OR  LIMIT-TO ( PUBYEAR ,  2016 )  OR  LIMIT-TO ( PUBYEAR ,  2015 )  OR  LIMIT-TO ( PUBYEAR ,  2014 )  OR  LIMIT-TO ( PUBYEAR ,  2013 )  OR  LIMIT-TO ( PUBYEAR ,  2012 )  OR  LIMIT-TO ( PUBYEAR ,  2011 )  OR  LIMIT-TO ( PUBYEAR ,  2010 ) ) | **652** |

CEE Database of Evidence Reviews (CEEDER)[https://environmentalevidence.org/ceeder/]

| **Search field** | **Search terms** | **Collection** | **Review Type** | **Hits** |
| --- | --- | --- | --- | --- |
| Title/Abstract/Keywords | (tax) OR (levy) OR (subsidy) OR (incentive) OR (voucher) OR (discount) | Climate Change | Evidence Overviews | 0 |
| Title/Abstract/Keywords | (tax) OR (levy) OR (subsidy) OR (incentive) OR (voucher) OR (discount) | Climate Change | Evidence Reviews | **16** |

Epistemonikos [https://www.epistemonikos.org/en/]

| **Search terms** | **Review type** | **Hits** |
| --- | --- | --- |
| (title:(tax OR taxes OR taxation OR subsidy OR subsidies OR levy OR levies OR voucher OR vouchers OR discount OR discounts OR incentive OR incentives) OR abstract:(tax OR taxes OR taxation OR subsidy OR subsidies OR levy OR levies OR voucher OR vouchers OR discount OR discounts OR incentive OR incentives)) AND (title:(food OR sugar OR fat OR salt OR meat OR fruit OR vegetables OR beverage OR beverages OR drin OR drinks OR soda OR sodas) OR abstract:(food OR sugar OR fat OR salt OR meat OR fruit OR vegetables OR beverage OR beverages OR drin OR drinks OR soda OR sodas) OR abstract:(food OR sugar OR fat OR salt OR meat OR fruit OR vegetables OR beverage OR beverages OR drin OR drinks OR soda OR sodas) OR abstract:(food OR sugar OR fat OR salt OR meat OR fruit OR vegetables OR beverage OR beverages OR drin OR drinks OR soda OR sodas)) | Broad synthesis/Scoping Review | 17 |
| (title:(tax OR taxes OR taxation OR subsidy OR subsidies OR levy OR levies OR voucher OR vouchers OR discount OR discounts OR incentive OR incentives) OR abstract:(tax OR taxes OR taxation OR subsidy OR subsidies OR levy OR levies OR voucher OR vouchers OR discount OR discounts OR incentive OR incentives)) AND (title:(food OR sugar OR fat OR salt OR meat OR fruit OR vegetables OR beverage OR beverages OR drin OR drinks OR soda OR sodas) OR abstract:(food OR sugar OR fat OR salt OR meat OR fruit OR vegetables OR beverage OR beverages OR drin OR drinks OR soda OR sodas) OR abstract:(food OR sugar OR fat OR salt OR meat OR fruit OR vegetables OR beverage OR beverages OR drin OR drinks OR soda OR sodas) OR abstract:(food OR sugar OR fat OR salt OR meat OR fruit OR vegetables OR beverage OR beverages OR drin OR drinks OR soda OR sodas)) | Systematic Review | 152 |
| (title:(tax OR taxes OR taxation OR subsidy OR subsidies OR levy OR levies OR voucher OR vouchers OR discount OR discounts OR incentive OR incentives) OR abstract:(tax OR taxes OR taxation OR subsidy OR subsidies OR levy OR levies OR voucher OR vouchers OR discount OR discounts OR incentive OR incentives)) AND (title:(environmental OR "climate change" OR "climatic change" OR greenhouse OR pollution OR "carbon footprint" OR biodiversity OR "global warming") OR abstract:(environmental OR "climate change" OR "climatic change" OR greenhouse OR pollution OR "carbon footprint" OR biodiversity OR "global warming")) | Broad synthesis/Scoping Review | 9 |
| (title:(tax OR taxes OR taxation OR subsidy OR subsidies OR levy OR levies OR voucher OR vouchers OR discount OR discounts OR incentive OR incentives) OR abstract:(tax OR taxes OR taxation OR subsidy OR subsidies OR levy OR levies OR voucher OR vouchers OR discount OR discounts OR incentive OR incentives)) AND (title:(environmental OR "climate change" OR "climatic change" OR greenhouse OR pollution OR "carbon footprint" OR biodiversity OR "global warming") OR abstract:(environmental OR "climate change" OR "climatic change" OR greenhouse OR pollution OR "carbon footprint" OR biodiversity OR "global warming")) | Systematic Review | 51 |
|  |  | **229** |

Overton

Restricted to: Published since 1 Jan 2010, document type: publication. Searched 15^th^ November 2022

| **Search terms** | **Hits** |
| --- | --- |
| abstract: (“food” OR “foods” OR “drink” OR “drinks” OR "beverage" OR "beverages" OR “fat*” OR “salt*” OR “sugar*” OR “fruit*” OR “vegetable*” OR “meat*” OR “dairy” OR "confectionery" OR “confectionary” OR "sweet*") AND (“subsidy” OR “subsidies” OR “subsidise*” OR “subsidize*” OR “tax*” OR “levy” OR “levie*” OR “tariff*” OR “duty” OR “duties” OR "coupon*" OR "voucher*" OR “excise” OR “ad valorem” OR “value-added”) AND ("policy" OR "policies" OR "strategy" OR "strategies" OR “framework” OR “measure*” OR “initiative*” OR “scheme*”) | **1,127** |

1. Conway JR, Lex A, Gehlenborg N. UpSetR: an R package for the visualization of intersecting sets and their properties. Bioinformatics. 2017 Sep 15;33(18):2938-2940. doi: 10.1093/bioinformatics/btx364. PMID: 28645171; PMCID: PMC5870712.
